# Safe and effective off-the-shelf immunotherapy based on CAR.CD123-NK cells for the treatment of acute myeloid leukaemia

**DOI:** 10.1186/s13045-022-01376-3

**Published:** 2022-11-05

**Authors:** Simona Caruso, Biagio De Angelis, Francesca Del Bufalo, Roselia Ciccone, Samantha Donsante, Gabriele Volpe, Simona Manni, Marika Guercio, Michele Pezzella, Laura Iaffaldano, Domenico Alessandro Silvestris, Matilde Sinibaldi, Stefano Di Cecca, Angela Pitisci, Enrico Velardi, Pietro Merli, Mattia Algeri, Mariachiara Lodi, Valeria Paganelli, Marta Serafini, Mara Riminucci, Franco Locatelli, Concetta Quintarelli

**Affiliations:** 1 Department of Oncology-Haematology, and Cell and Gene Therapy, Bambino Gesù Children Hospital, IRCCS, Rome, Italy; 2 Department of Molecular Medicine, Sapienza University of Rome, Rome, Italy; 3 Research Laboratories, Bambino Gesù Children’s Hospital, IRCCS, 00146 Rome, Italy; 4 Department of Pediatrics, Tettamanti Research Center, Fondazione MBBM/San Gerardo Hospital, University of Milano-Bicocca, Monza, Italy; 5 Department of Life Science and Public Health, Università Cattolica del Sacro Cuore, Rome, Italy; 6 Department of Clinical Medicine and Surgery, University of Naples Federico II, Naples, Italy

## Abstract

**Background:**

Paediatric acute myeloid leukaemia (AML) is characterized by poor outcomes in patients with relapsed/refractory disease, despite the improvements in intensive standard therapy. The leukaemic cells of paediatric AML patients show high expression of the CD123 antigen, and this finding provides the biological basis to target CD123 with the chimeric antigen receptor (CAR). However, CAR.CD123 therapy in AML is hampered by on-target off-tumour toxicity and a long “vein-to-vein” time.

**Methods:**

We developed an off-the-shelf product based on allogeneic natural killer (NK) cells derived from the peripheral blood of healthy donors and engineered them to express a second-generation CAR targeting CD123 (CAR.CD123).

**Results:**

CAR.CD123-NK cells showed significant anti-leukaemia activity not only in vitro against CD123^+^ AML cell lines and CD123^+^ primary blasts but also in two animal models of human AML-bearing immune-deficient mice. Data on anti-leukaemia activity were also corroborated by the quantification of inflammatory cytokines, namely granzyme B (Granz B), interferon gamma (IFN-γ) and tumour necrosis factor alpha (TNF-α), both in vitro and in the plasma of mice treated with CAR.CD123-NK cells.

To evaluate and compare the on-target off-tumour effects of CAR.CD123-T and NK cells, we engrafted human haematopoietic cells (hHCs) in an immune-deficient mouse model. All mice infused with CAR.CD123-T cells died by Day 5, developing toxicity against primary human bone marrow (BM) cells with a decreased number of total hCD45^+^ cells and, in particular, of hCD34^+^CD38^−^ stem cells. In contrast, treatment with CAR.CD123-NK cells was not associated with toxicity, and all mice were alive at the end of the experiments. Finally, in a mouse model engrafted with human endothelial tissues, we demonstrated that CAR.CD123-NK cells were characterized by negligible endothelial toxicity when compared to CAR.CD123-T cells.

**Conclusions:**

Our data indicate the feasibility of an innovative off-the-shelf therapeutic strategy based on CAR.CD123-NK cells, characterized by remarkable efficacy and an improved safety profile compared to CAR.CD123-T cells. These findings open a novel intriguing scenario not only for the treatment of refractory/resistant AML patients but also to further investigate the use of CAR-NK cells in other cancers characterized by highly difficult targeting with the most conventional T effector cells.

**Supplementary Information:**

The online version contains supplementary material available at 10.1186/s13045-022-01376-3.

## Background

Acute myeloid leukaemia (AML) is a malignant clonal disease characterized by the presence of recurrent genetic alterations, which result in the inhibition of cell differentiation and the proliferation or accumulation of blasts. Despite the remarkable improvements in the treatment of AML achieved in recent decades, a significant proportion of patients are refractory to first-line treatment or experience relapse, portending a poor outcome [1].

In recent years, immunotherapy approaches have been widely developed to treat patients with acute leukaemia. In particular, one of the most successful treatments is based on genetically engineered T cells expressing chimeric antigen receptors (CARs), where T cell specificity is redirected towards cell surface antigens expressed on leukaemic cells. While several CAR T cell products are approved by the US Food and Drug Administration (FDA) and European Medicines Agency (EMA) for the treatment of relapsed or refractory (r/r) B cell lymphoid malignancies or multiple myeloma [2], there is no product currently approved for the treatment of AML or envisaged to reach the last stage of market authorization in the next few years. One major hurdle for targeting AML with immunotherapy is represented by the identification of an optimal surface target antigen, with selective high expression on leukaemic blasts and negligible expression on healthy cells, particularly those in the haematopoietic compartment. Several antigens (including CD33, CD123, CLL1, CD244, TIM3 and CD7), known to be overexpressed on AML leukaemic stem cells (LSCs), have been extensively evaluated in preclinical models [3] and early clinical settings [4].

Interleukin-3 receptor alpha chain (IL-3Rα), more commonly referred to as CD123, has become an attractive therapeutic target because it is widely expressed in several haematological malignancies, including AML and B cell precursor acute lymphoblastic leukaemia (BCP-ALL). In particular, it has been reported that CD123 is expressed on both LSCs and more differentiated leukaemic blasts [5, 6]; in addition, a correlation between CD123 expression on AML LSCs and poor prognosis due to resistance to traditional chemotherapy has been found [7]. Adoptive cell transfer with CAR T cells engineered to recognize CD123 cells has been tested in several clinical trials for patients with AML (NCT04230265, NCT03766126, NCT04678336) [4, 8]; however, no conclusive data on safety and efficacy are available thus far, and the improvement of the approach to achieve complete remission (CR) while preventing potential toxicity is still an unmet clinical need. Indeed, several preclinical studies have shown that CD123 is also expressed on healthy haematopoietic and endothelial cells, although at a lower level than that observed on AML blasts [9, 10]. In particular, the specific CD123 expression pattern on haematopoietic progenitor cells has led to the evaluation of the potential application of CAR.CD123-T cells [11] as a myeloablative conditioning regimen for haematopoietic stem cell transplantation (HSCT) instead of chemo-/radiotherapy [12].

In addition to these peculiar difficulties in targeting a well-known antigen on AML blasts, the most common hurdles associated with CAR T cell approaches, namely (1) cytokine release syndrome (CRS); (2) immune effector cell-associated neurotoxicity syndrome (ICANS) [13]; and (3) CAR-associated haemophagocytic lymphohistiocytosis [14], must be taken into consideration, as well as the expensive and time-consuming production of these cells, leading to “vein-to-vein” times that are often unfeasible in patients with r/r AML [15]. Moreover, in the setting of r/r AML patients, previous treatment with high-intensity chemotherapy regimens and significant contamination of leukaemia blasts in peripheral blood (PB) could lead to a poor quality of the autologous apheresis product, affecting the success rate of the manufacturing process [16].

In light of these consideration, the use of third-party, off-the-shelf products, such as those based on allogeneic natural killer (NK) cells genetically modified with a CAR targeting LSCs, represents an ideal option.

NK cells belong to the innate immune system and play a pivotal role as first-line defenders to recognize and kill virally infected or tumour cells. Moreover, NK cells provide an attractive alternative to T cells for CAR-cell therapy, as they do not cause graft-versus-host disease (GvHD) [17]. The cytotoxic activity of NK cells is carried out by the direct interaction of their activating receptors (i.e. NKG2D, NKp30, NKp46, 2B4 and CD16) associated with their coreceptors (ex. DAP10, CD3ζ and FcεRIγ) with ligands on the surface of target cells [18] [19]. In addition to the native NK receptors, NK-cell antitumour activity can also be mediated through antibody-dependent cell-mediated cytotoxicity (ADCC), with CD16 (FcRγIII) recognizing the Fc portion of tumour-bound antibodies, inducing the release of cytotoxic factors and cytokines and, ultimately, tumour cell lysis [20]. All these features of NK-cell functions, in the absence of alloreactivity, underline their high clinical value. In the setting of AML, the ability of NK cells to recognize and kill AML blasts, based on the expression of NK-cell activating ligands on leukaemic cells, has been largely described [21].

As for T cells, NK cells can be genetically modified to express CARs that recognize tumour-associated cell surface antigens to mediate lysis of cancer cells. In particular, we have already demonstrated that PB-derived CAR-NK cells (PB.CAR-NK cells) can be generated and expanded with a robust, feeder-free, bovine serum-free protocol for the manufacturing of an off-the-shelf product [22]. While several clinical trials are available for CAR T cells, clinical data regarding CAR-NK cells are still limited [23], and most of them are based on irradiated NK-cell lines (e.g. NK-92 or the newly generated cell line NK-101) [24]. Promising clinical results have been reported by Liu and colleagues, who infused NK cells derived from a single umbilical cord unit genetically modified to coexpress anti-CD19 CAR and interleukin-15 to treat patients with r/r B cell lymphoproliferative diseases [25].

In light of these findings, we have developed an off-the-shelf cell product consisting of engineered NK cells expressing a second-generation CAR directed against CD123 (CAR.CD123-NK cells), designed to enhance NK-cell antitumour effects in AML patients and to reduce unwanted toxicity. We demonstrated that CAR.CD123-NK cells exert potent antigen-dependent activation and cytotoxicity both in in vitro and in in vivo models. In addition, to the best of our knowledge, for the first time, we were able to prove that, in contrast to CAR.CD123-T cells, the use of CAR.CD123-NK cells do not elicit either severe haematopoietic toxicity in a humanized mouse model or endothelial injury in a human endothelial murine model.

## Methods

### Human samples

Primary AML and healthy donor (HD) bone marrow (BM) samples were obtained from the Bambino Gesù Children’s Hospital (OPBG), Rome, Italy. Written informed consent was obtained from each donor. The studies were approved by the Institutional Ethical Committee N 2553_OPBG_2021. BM stem cells (BMSCs) were isolated and cultured as previously described to obtain mesenchymal stromal cells (MSCs) for the generation of the endothelial model [26].

### Plasmid construction and retrovirus production

The retroviral plasmid was designed to carry the cassette of a second-generation CAR with specificity for CD123. A single-chain variable fragment (scFv), derived from a murine 7G3 antibody, was linked via an optimized human CD8 hinge-transmembrane domain to the costimulatory domains 4-1BB (CD137) and CD3-ζ. As a trackable marker, we added a peptide derived from the human phosphoglycoprotein CD34 (ΔCD34) [27].

Retroviral supernatant was generated in 293 T cells [28] and the viral particles (VPs) was quantified using a Retro-X™ qRT-PCR Titration Kit (Takara). NK and T cells were transduced using 10^9^ VPs/0.5 × 10^6^ cells.

### Leukaemia cell lines

The human leukaemia cell lines THP-1, OCI-AML3, and MOLM-13 were obtained from ATCC (American Type Culture Collection, Manassas, Virginia, USA) and cultured in RPMI-1640 supplemented with 10% foetal bovine serum (HyClone, Thermo Scientific, Pittsburgh, PA), 2 mM Gluta-Max (Invitrogen, California, USA) and 100 U/mL penicillin/streptomycin (EuroClone, Italy). Cells were maintained in a humidified atmosphere containing 5% CO_2_ at 37 °C and were routinely tested for mycoplasma and for surface expression of target antigens. For selected in vitro and in vivo studies, cell lines were transduced with a retroviral vector encoding eGFP-firefly luciferase (eGFP-FFLuc) to enable detection by bioluminescence imaging (FFLuc). HUVECs were purchased from Cambrex Corporation and grown in a Clonetics EGM-2 Bullet Kit following the manufacturer’s instructions.

### Generation and expansion of CAR-modified NK and CAR T cells

Peripheral blood mononuclear cells (PBMCs) were isolated by the density-gradient technique as previously described [27] from HD buffy coat (BC) or leukapheresis obtained from OPBG, Rome, Italy. The HD signed a written informed consent form, in accordance with rules set by the Institutional Review Board of OPBG (Approval of Ethical Committee N. 2553_OPBG_2021).

NK cells (CD3^−^CD56^+^) were isolated with a NK isolation Kit (Miltenyi Biotec, Inc., San Diego, CA, USA) and expanded in vitro in the presence of recombinant human interleukin 2 (IL2, 500 U/ml; R&D; USA) and NK MACS medium (Miltenyi Biotec, Germany) [22]. T cells were activated with OKT3 (1 μg/ml, Thermo Fisher Scientific, USA) and anti-CD28 (1 μg/ml, BD Biosciences, USA) monoclonal antibodies (mAbs) with a combination of recombinant human (rh) interleukin-7 (rh-IL7, 10 ng/ml; Bio-Techne; USA) and rh-IL15 (5 ng/ml; Bio-Techne; USA) [29]. After 3 days, T and NK cells were transduced with a γ-retroviral supernatant in 24-well plates precoated with RetroNectin (Takara-Bio; Japan). T and NK lymphocytes were expanded in the presence of cytokines; T cells were expanded in completed medium containing 45% RPMI 1640 and 45% Click's medium (Sigma‒Aldrich, Co.; USA), while NK cells were expanded in NK MACS medium (Miltenyi Biotec, Germany), which was replenished twice a week.

### Phenotypic analysis

The anti-human antibodies used for flow cytometry of HD and AML patient primary cells are listed in the Additional file 1. In all analyses, the population of interest was gated based on forward vs. side scatter characteristics followed by singlet gating, and live cells were gated using 7-amino-actinomycin D (7-AAD) (BD Biosciences, Italy). The expression of CAR.CD123 on T and NK cells was detected by an anti-CD34 (cat. no FAB7227P, R&D Systems) and was evaluated over time, as indicated, in association with CD56- or CD3-specific mAbs and the isotype control. Flow cytometry analyses were performed on a FACSCanto II or LSRFortessa (BD Biosciences), and the data were analysed using FACSDiva software (BD Biosciences).

### Coculture assay

For in vitro functional analysis, un-transduced NK cells (NT-NK) and CAR.CD123-NK cells were plated at Day 0 with an effector/target cell ratio (E:T) of 1:1 (0.25 × 10^6^ cells/well). The antitumour effect was evaluated in a flow cytometry assay after 6 days, assessing residual live 7AAD- CD123^+^ tumour cells. The coculture media was collected after 24 h to evaluate the secretion of cytokines, namely granzyme B (Granz B), interferon gamma (IFN-γ) and tumour necrosis factor alpha (TNF-α), by immunoassay in a microfluidic Simple Plex cartridge ELLA (Bio-Techne; R&D Systems, Minneapolis).

### Colony formation units

CD34^+^ cells were isolated from bone marrow mononuclear cells (BMMNCs) of HD samples using the CD34 Ultrapure Microbead kit (Miltenyi Biotec, Germany). HD CD34^+^ cells were first incubated with media alone or with NT-NK, T-NT cells, CAR.CD123-NK or CAR.CD123-T cells at an E:T ratio of 1:1 in StemSpan Serum-Free Expansion Medium (SFEM, Stem Cell Technologies) for 4 h.

Following incubation, the cell suspension was added to a semisolid methylcellulose-based medium (Methocult opti H3404, Stem Cell Technologies, Vancouver, BC, Canada) and plated into 3-cm tissue culture dishes. After 14 days, colonies were counted using established criteria according to the manufacturer’s instructions and scored using an inverted microscope (Zeiss 4 ×).

### Xenogenic in vivo leukaemia models

In vivo experiments were approved by the Italian Health Ministry (N°88/2016-PR). NOD/SCID IL-2Rγnull (NSG) xenograft mice were infused with THP-1 cells to assess the antitumour effect of CAR-NK cells and CAR T cells in vivo. Mouse experiments were conducted in compliance with the international, EU and national ethical requirements and were approved by the Italian Health Ministry (n. 238/2022-PR 12/4/2022). NSG mice (6-week-old female mice; The Jackson Laboratory, USA) were inoculated with firefly luciferase-labelled THP-1 cells (THP-1-FF.Luc) (5 × 10^4^/mouse) on Day -3. Mice were then injected i.v. twice (on Day 0 and Day 7) with 5 × 10^6^ NT-T, CAR.CD123-T, NT-NK or CAR.CD123-NK cells and subjected to weekly bioluminescence imaging (IVIS System, Perkin Elmer, USA). Signal quantitation of photons/second was performed as previously described [30].

Six-week-old NOD.Cg-Prkdcscid Il2rgtm1Sug Tg (CMV-IL2/IL15) (hNOG-IL15) female mice were purchased from Taconic laboratories. [31] hNOG-IL15 mice were engrafted with THP-1-FF.Luc cells (5 × 10^4^/mouse) on Day -3 and treated with a single *i.v.* dose of 20 × 10^6^ NT-NK cells or CAR.CD123-NK cells on Day 0. The expansion of effector cells was monitored by PB collection and analysed by flow BD LSR Fortessa X-20 cytometry. Data were analysed using FACSDiva software (BD Biosciences, Italy). All mice were killed according to the protocol when either moribund or upon the development of hind-limb paralysis.

### Humanized murine model

Six-week-old hGM-CSF/hIL3 NOG female mice were purchased from Taconic Laboratories and engrafted with human umbilical cord blood-derived CD34^+^ haematopoietic stem cells (HSCs) [32]. Ten weeks after hCD45^+^ engraftment, mice were quality checked for human leukocyte reconstitution by flow cytometry. Only mice reaching ≥ 25% hCD45^+^ cells were used in the experiments. Humanized mice were intravenously infused with 10 × 10^6^ NT-T cells or CAR.CD123-T cells and NT-NK or CAR.CD123-NK cells. On Day 7 following effector cell infusion, mice were killed, and BM, spleen, and PB samples were FACS-analysed for changes in multilineage engraftment.

### On-target off-tumour effect in an in vivo endothelial mouse model

The in vivo endothelial mouse model was established as follows: 1 × 10^6^ hMSCs were mixed with an equal number of HUVECs (Cambrex) and suspended in 1 ml of growth factor-reduced Matrigel (BD Biosciences). Either NSG or hNOG-IL15 xenograft mice were injected subcutaneously in the back with 0.7 ml of suspension on Day 0 [33]. NSG mice bearing endothelial transplants were treated with either NT-T or CAR.CD123-T cells on Day 15, while hNOG-IL15 mice bearing the endothelial transplant were treated with either NT-NK or CAR.CD123-NK cells. The endothelial transplants were harvested on Day 30 and analysed for microvessel density as previously described by Melero-Martin et al., 2008 [34]. Orthotopic transplants were processed as described in the Additional file 1, and IHC studies were performed to analyse both CD123 expression on the vessel surface and CD45^+^ infiltrated effector cells (more details in Additional file 1: Supplementary Materials).

Haematoxylin/eosin and IHC staining were analysed by Zeiss microscope at an original magnification of 10X and 20X, respectively. Scale bars represent 50 or 100 µm.

### Statistical analyses

Statistical analyses and significance were determined using GraphPad Prism v7.01 or v8.2.1 software (GraphPad Software Inc. LA Jolla, CA). Based on the results of the normality tests, a paired Student’s t test, unpaired or paired parametric (two-sided) Student’s t test, unpaired nonparametric (two-sided) Student’s t test (Mann–Whitney test), ordinary two-sided one-way ANOVA (Tukey’s multiple comparisons test) or two-sided log-rank (Mantel–Cox) was used, where appropriate, for comparing differences between groups. A log-rank test was also used to calculate significant differences between survival curves. All graphed data, except for survival curve data, are presented as the median or mean ± standard error of the mean (SEM). In all cases, a *p* value of ≤ 0.05 was considered significant. Exact *p* values are given whenever suitable. Wilcoxon or Mann–Whitney nonparametric tests were employed. Statistical significance (*p* value: *p < 0.05, **p ≤ 0.01, ***p ≤ 0.001) is indicated.

## Results

### CD123 is a relevant target for leukaemic stem cells of patients with AML

**Fig. 1:**
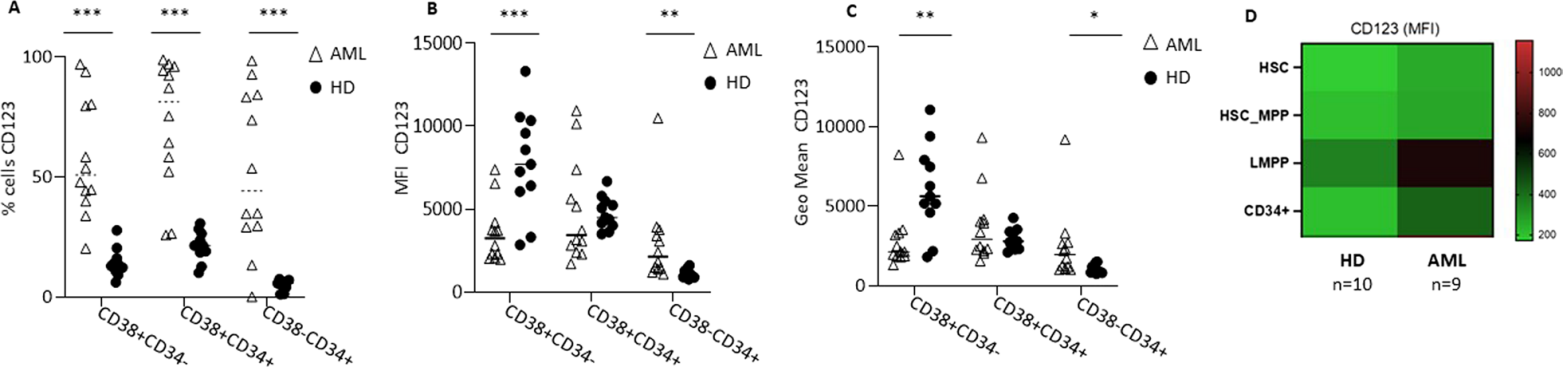
CD123 is highly expressed on primary blasts collected from paediatric AML patients. **A** Percentage of CD123 expression levels in primary BM cells obtained from AML patients (triangles) and HD (circles) were assessed by flow cytometry analysis, across 3 main haematopoietic cell subpopulations, namely the mature CD38^+^CD34^−^ cells, the intermediate CD38^+^ CD34^+^ cells, and the most immature CD38^−^ CD34^+^ stem cells. The gating strategy is shown in Additional file 1: Fig. S1. **B** Median fluorescence intensity (MFI) for CD123 on AML primary blasts or BM-derived cells from HDs. MFI was adjusted for cell size by dividing MFI by the forward scatter (FSC). **C** Geometric mean fluorescence intensities (GeoMean) of CD123 on AML primary blasts or BM-derived cells from HDs. Data are shown as average ± SD. *p < 0.05, **p < 0.01, ***p ≤ 0.001. **D** The heatmap shows the CD123 MFI detected on HSC precursors from BM of AML patients and HDs

To prove that CD123 is a suitable AML cell surface target for CAR-cell therapy, we investigated its differential expression on BM-derived cells either from paediatric patients at the diagnosis of AML (*n* = 12) or from HDs (*n* = 11). We proved that the CD123 antigen was highly expressed across the different stages of myeloid leukaemia differentiation, including the most mature CD38^+^ CD34^−^ leukaemic cells (mean of %CD123 positive cells: 57.8 ± 24.5, Fig. 1A, Additional file 1: Fig. S1), the intermediate CD38^+^ CD34^+^ leukaemic cells (mean of %CD123 positive cells: 72.37 ± 26.9, Fig. 1A, Additional file 1: Fig. S1), and the undifferentiated CD38^−^ CD34^+^ leukaemic cells (mean of %CD123 positive cells: 52.3 ± 33.2, Fig. 1A, Additional file 1: Fig. S1). Notably, CD123 was expressed in a significantly lower percentage of haematopoietic cells derived from the BM of HDs for all investigated myeloid maturation cell stages (Fig. 1A, Additional file 1: Fig. S2). In particular, we observed that CD123^+^ mature haematopoietic cells (CD38^+^ CD34^−^) from HDs, although they were detected with low frequency, were characterized by a higher MFI and GeoMean than AML cells (Fig. 1B, *p* < 0.001 and 1C, *p* < 0.001), whereas undifferentiated CD38^−^ CD34^+^ leukaemic cells showed a higher CD123 MFI and GeoMean than HD haematopoietic cells (Fig. 1B, *p* = 0.003 and 1C, *p* = 0.04). A more accurate characterization of more immature haematopoietic progenitors showed that CD123 expression was observed up to lymphomyeloid-committed progenitor (LMPP), haematopoietic stem cells and multipotent progenitors (HSC-MPPs) and, last, haematopoietic stem cells (HSC), with a significantly higher percentage of CD123^+^ cells in AML BM samples than in BM samples from HDs (Fig. 1D).

### CAR.CD123-NK cells exert strong cytotoxic activity against CD123^+^ tumour cell lines

**Fig. 2:**
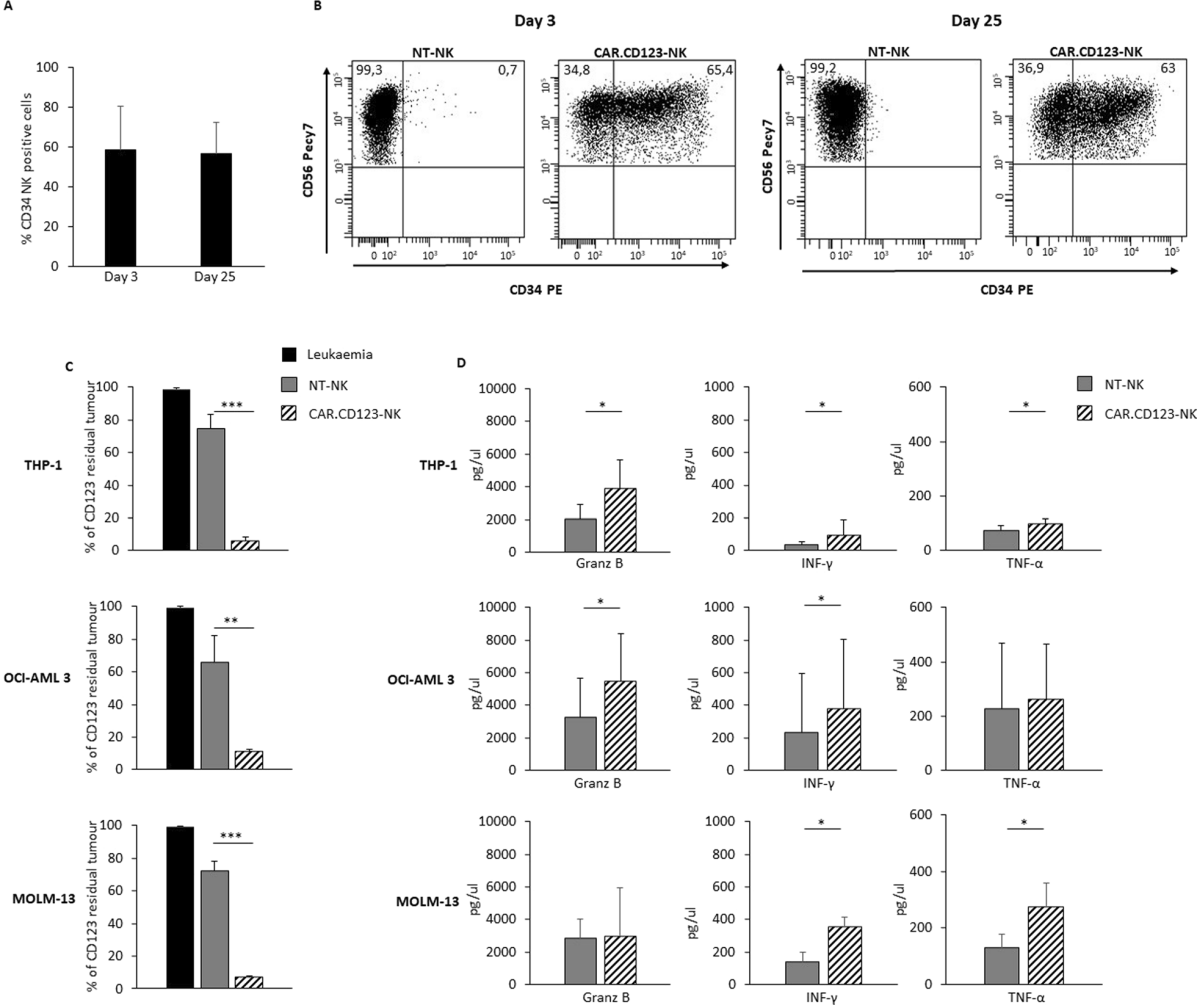
CAR.CD123-NK cells exert a significant cytotoxicity against CD123^+^ AML cells. A–B CAR.CD123 is stably expressed on NK cells over extensive in vitro culture (evaluated up to 25 days). **A** The CAR.CD123 expression on NK cells from 9 different HDs was monitored at Day 3 and at Day 25 after transduction and is represented as average ± SD (**B**) Representative FACS analysis of CAR.CD123 expression in NK cells at Day 3 and Day 25 after transduction is shown. CAR expression was assessed by the use of anti-CD34 for the CAR detection in combination with anti-CD56 for NK detection. **C** Long-term 6-day cocultures were performed in 9 independent experiments, in which CD123^+^ leukaemia cell lines (namely, THP-1, OCI-AML3, and MOLM-13) (black bars) were cocultured with either NT-NK (grey bars) or CAR.CD123-NK cells (line black bars) derived from 9 independent HDs at the E:T ratio of 1:1. Data are shown as average ± SD. **D** Granz B, IFN-γ and TNF-α were measured by ELISA assay in 24 h culture supernatant of NT-NK (grey bars) or CAR.CD123-NK cells (line black bars) in response to CD123.^+^ leukaemia cell lines (namely, THP-1, OCI-AML3, and MOLM-13). Cytokine analysis was performed in 4 independent experiments, and data are shown as average ± SD. *p < 0.05, **p < 0.01, ***p < 0.001

Considering the high expression of CD123 on AML blasts, we generated a retroviral vector encoding a second-generation CAR.CD123.41bb.CD3z (Additional file 1: Fig. S3A) to genetically modify primary NK cells purified from either the BC or leukapheresis of HDs (PB-NK). Activated NK cells were transduced on Day + 4 and expanded in vitro up to 30 days in a feeder-free approach [22]. The average CAR.CD123 transduction efficiency on CAR.CD123-NK cells was 58.8% ± 21.9 (Day 7; *n* = 8; range: 32.4–94%), with CAR expression remaining stable over time (evaluated up to Day 25 of culture) (Fig. 2A–B). Notably, we observed that CAR.CD123 was efficiently expressed on both in vitro-expanded CD56^+^CD16^−^ and CD56^+^CD16^+^ NK cells (Additional file 1: Fig. S3B). To assess the cytotoxic activity of CAR.CD123-NK cells against CD123-positive (CD123^+^) target cells, CAR.CD123-NK cells and NT-NK cells were coincubated with the THP-1, OCI-AML-3 and MOLM-13 cell lines (*n* = 9; Fig. 2C) at an effector/target (E:T) ratio of 1:1 in the absence of cytokine provision. The anti-leukaemia activity was evaluated with a 6-day long-term coculture, and the residual CD123^+^ leukaemic cells were evaluated by flow cytometry. CAR.CD123-NK cells displayed a significantly higher killing of all the leukaemic cell lines than NT-NK cells, which were not able to eliminate CD123^+^ leukaemic cells from the culture (Fig. 2C). Moreover, as shown in Fig. 2D, the anti-leukaemia activity data were supported by the release of inflammatory cytokines, namely Granz B, IFN-γ and TNF-α. Last, we proved the high specificity of CAR.CD123-NK-cell activity, since no anti-leukaemia activity or cytokine production was observed against CD123-negative KARPAS 299 cells (Additional file 1: Fig. S4A-B).

### Primary AML blasts are recognized and efficiently killed by CAR.CD123-NK cells in vitro

To corroborate the relevance of our findings, we tested the anti-leukaemia activity of CAR.CD123-NK cells against primary blasts obtained from paediatric AML BM samples.

**Fig. 3:**
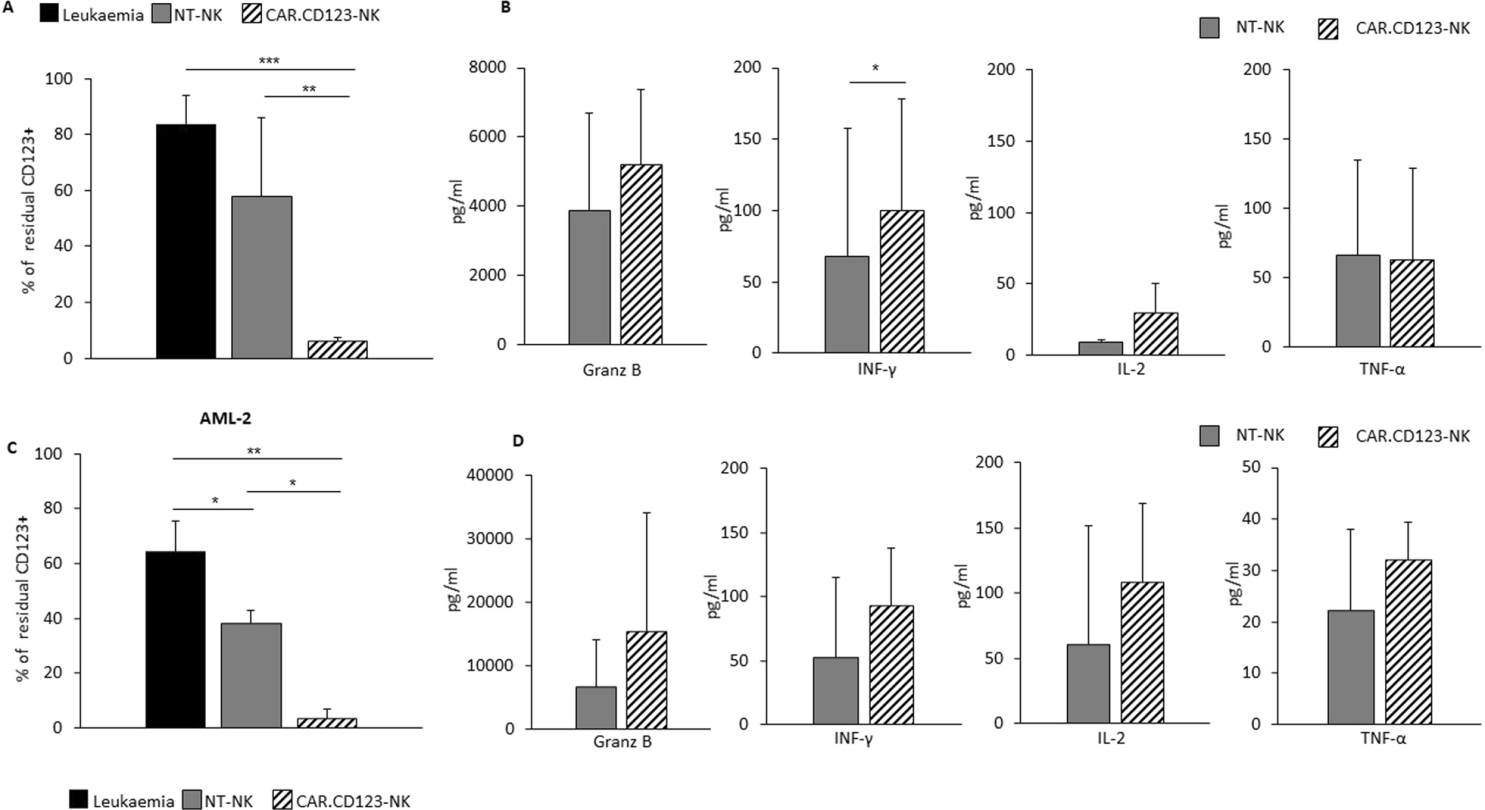
CAR.CD123-NK cells efficiently recognize and kill primary AML blasts. **A** Leukaemic cells obtained from BM samples of 3 AML patients (AML#1, AML#3, AML#4) at diagnosis (black bars representing leukaemic cells alone) were cocultured for 6 days with NT-NK (grey bar), or CAR.CD123-NK (line black bar) derived from a single HD. Coculture was performed at an E:T ratio of 1:1. Data of residual CD123^+^ leukaemic cells is shown as average ± SD. **B** Granz B, IFN-γ, IL-2 and TNF-α were measured by ELISA assay in 24-h culture supernatant of NT-NK (grey bars) or CAR.CD123-NK cells (line black bars) in response to CD123^+^ leukaemic cells. **C** Leukaemic CD123^+^ cells from BM sample of one single AML patient (AML #2; black bar represent leukaemic cells alone) were cocultured with NT-NK (grey bars) or CAR.CD19-NK (line black bars) derived from 3 independent HDs. Coculture was performed at the E:T ratio of 1:1. Data of residual total CD123^+^ leukaemic cells is shown as average ± SD. *p < 0.05. **D** Granz B, IFN-γ, IL-2 and TNF-α were measured by ELISA assay in 24 h culture supernatant of NT-NK (grey bars) or CAR.CD123-NK cells (line black bars) in response to CD123.^+^ leukaemic cells from AML#2 patient. *p < 0.05

To assess the consistency of the anti-leukaemia activity of CAR.CD123-NK cells independently from HLA-KIR haplotype mismatch, we used leukaemia blasts from 3 AML patients in long-term (6 days) coculture assays with CAR.CD123-NK cells derived from HDs and both patients and HDs were randomly identified, regardless of HLA mismatch. Figure 3A shows that CAR.CD123-NK cells were able to exert significant leukaemia cytotoxicity towards CD123^+^ blasts derived from all 3 AML patients (Additional file 1: Fig. S5), with minimal variability (CAR.CD123-NK; St Dv = 7.4). Nevertheless, as expected, NT-NK cells modestly, but not significantly, controlled the leukaemia, with a high observed variability (NT-NK; St Dv = 28.4). Proinflammatory cytokines (namely, Granz B, IL-2, IFN-γ and TNF-α) evaluated after 24 h of coculture confirmed the higher activation of CAR.CD123-NK cells than NT-NK cells after incubation with primary AML CD123^+^ blasts (Fig. 3B; Granz B, IFN-γ, TNF-α *p* < 0.05). To further explore the hypothesis of HLA-KIR haplotype mismatch-independent CAR activity of CAR.CD123-NK cells, we then challenged them with CAR.CD123-NK cells derived from 3 randomly chosen HDs against leukaemic blasts from a single AML patient (AML#2, Fig. 3C–D and Additional file 1: Fig. S5). Additionally, in this case, we demonstrated that CAR.CD123 significantly increased the recognition and cytotoxicity of NK cells against CD123^+^ leukaemic cells (38.1% ± 4.8 NT-NK cells vs. 3.6% ± 3.4 CAR-NK cells of CD123^+^ residual leukaemic cells; *p* > 0.05).

**Fig. 4:**
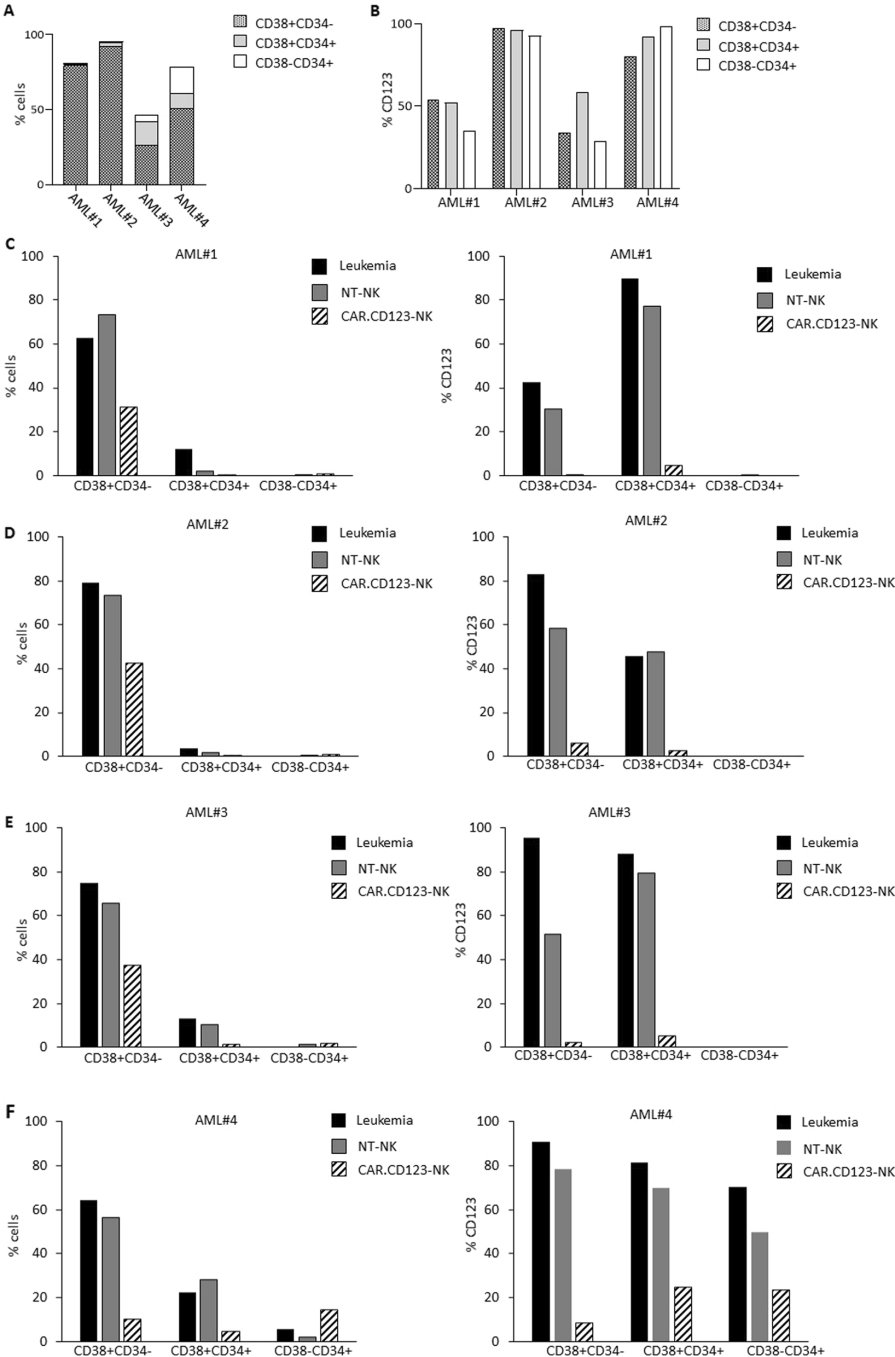
CAR.CD123-NK cells show a strong anti-leukaemia activity against the most relevant AML haematopoietic maturation subsets. **A** Percentage of CD38^+^CD34^−^ (dotted bar), CD38^+^CD34^+^ (light grey bar) and CD38^−^CD34^+^(white bar) cells obtained from BM samples of AML #1, AML #2, AML#3 and AML#4 were analysed by flow cytometry at thawing. **B** Percentage of CD123^+^ cells in the different haematopoietic maturation subsets, namely CD38^+^CD34^−^ (dotted bar), CD38^+^CD34^+^ (light grey bar) and CD38^−^CD34^+^(white bar) analysed at thawing of AML #1, AML #2, AML#3 and AML#4 BM samples (analysis performed before the in vitro culture of the cells). **C** AML #1, (**D**) AML #2, (**E**) AML#3 and (**F**) AML#4 BM cells have been independently analysed for the residual percentage of total CD38^+^CD34^−^, CD38^+^CD34^+^ and CD38^−^CD34^+^ cells (on the left) and for the residual percentage of CD123^+^ cells (on the right panel) after 6-day culture of leukaemic cells alone (black bars), leukaemic cells with NT-NK cells (grey bars) or with CAR.CD123-NK cells (black line bars) at the E:Tratio of 1:1

To further investigate the strength of our adoptive immunotherapy, we sought to evaluate whether CAR.CD123-NK cells were able to recognize and eliminate CD34^+^CD38^−^ AML cells, which are known to represent the leukaemia-initiating cell compartment characterized by high resistance to chemotherapy [35]. To this end, we examined in depth the anti-leukaemia activity of CAR.CD123-NK cells against all myeloid maturation subsets of the selected AML samples (Fig. 4A shows the percentage of CD38^+^CD34^−^, CD38^+^CD34^+^, CD38^−^CD34^+^ cell subsets in the 4 AML samples, and Fig. 4B reports the percentage of CD123^+^ cells for each mature myeloid subset).

In particular, we first confirmed that the culture conditions did not significantly affect the maturation stage or viability of any cell subset (Fig. 4C–F). Flow cytometry analyses highlighted that after 6 days of in vitro culture, all the myeloid maturation stages were preserved, as well as the percentage of CD123^+^ cells (data reported in Fig. 4A–B are for the myeloid maturation subtypes in AML specimens before the in vitro culture, whereas data reported in Fig. 4C–F are for the cell composition after 6 days of in vitro culture). Notably, we proved that CAR.CD123-NK cells significantly eradicated CD123^+^ cells in all CD38^+^CD34^−^, CD38^+^CD34^+^, and CD38^−^CD34^+^ cell subsets compared to the control conditions (leukaemia alone and NT-NK cells; Fig. 4C–F). Furthermore, blasts from AML#4, characterized by a significant percentage of the rare CD38^−^CD34^+^ population, were relevant to demonstrate that CAR.CD123-NK cells were able to reduce the percentage of CD123^+^ cells in in vitro culture (Fig. 4F).

### CAR.CD123-NK cells display robust anti-leukaemia activity in vivo

**Fig. 5:**
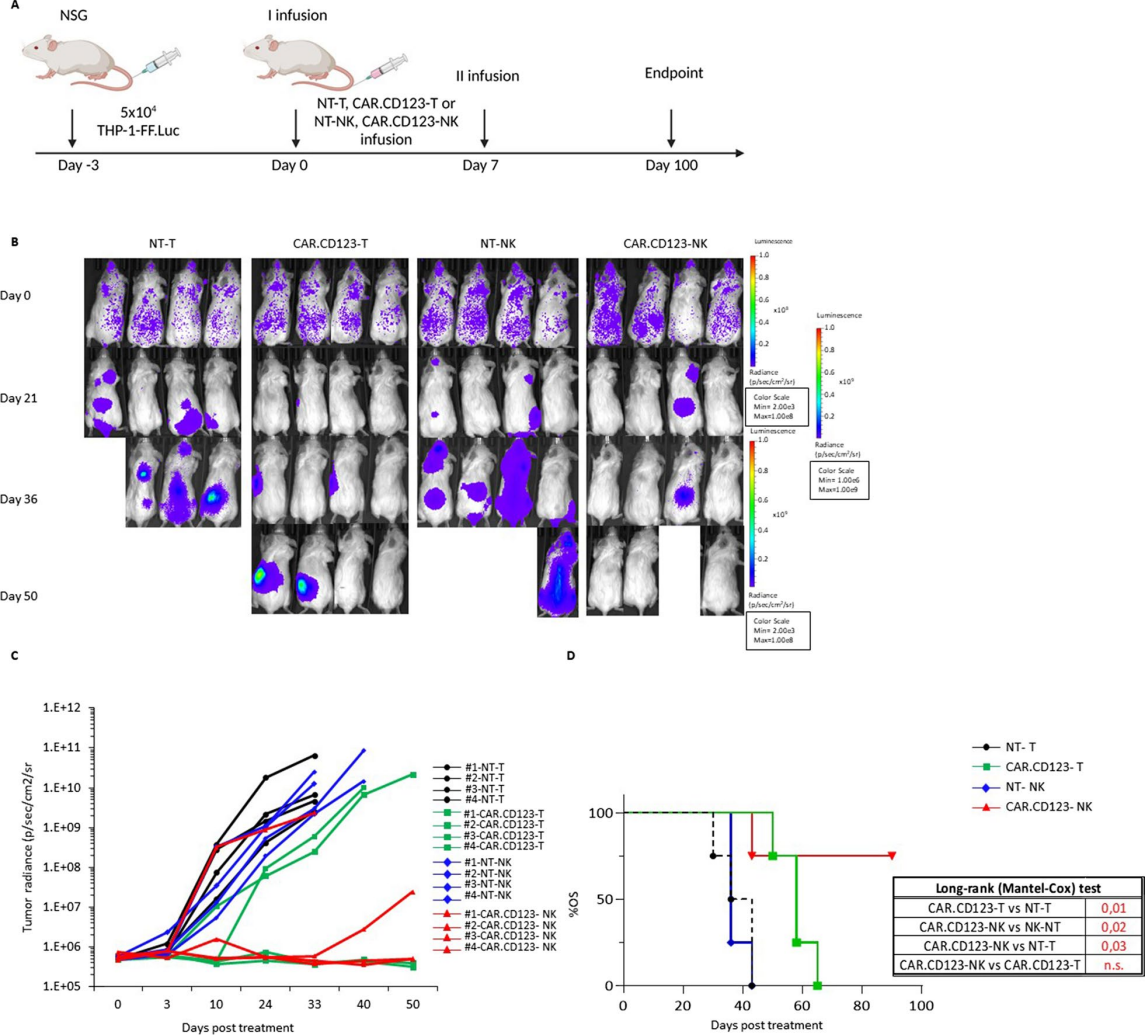
CAR.CD123-NK cells exert a significant anti-leukaemia activity in an AML xenograft NGS model. **A** Illustration of experimental setting in which NSG mice were systemically infused with THP-1-FF-Luc.GFP cells and, at the time of leukaemia engraftment, treated with NT-T, CAR.CD123-T, NT-NK and CAR.CD123-NK cells. **B** Time course of in vivo bioluminescence imaging of the treated NSG mice from Day 0 (Day of effector cells infusion) to Day 50. **C** Graph shows bioluminescence analysis of each leukaemia-bearing mouse treated with NT-T (black line with circles), CAR.CD123-T (green line with squares), NT-NK (blue line with pentagon) and CAR.CD123-NK (red line with triangles) cells. **D** One hundred days probability of overall survival (OS) of leukaemia-bearing mice treated with 2 consecutive adoptive transfers of NT-T (black line with circles), CAR.CD123-T (green line with squares), NT-NK (blue line with pentagon) and CAR.CD123-NK (red line with triangles) cells. On the right bottom, the statistical analysis of 100-Day OS of leukaemia-bearing mice treated with 2 consecutive adoptive transfers of NT-T, CAR.CD123-T, NT-NK, and CAR.CD123-NK cells

**Fig. 6:**
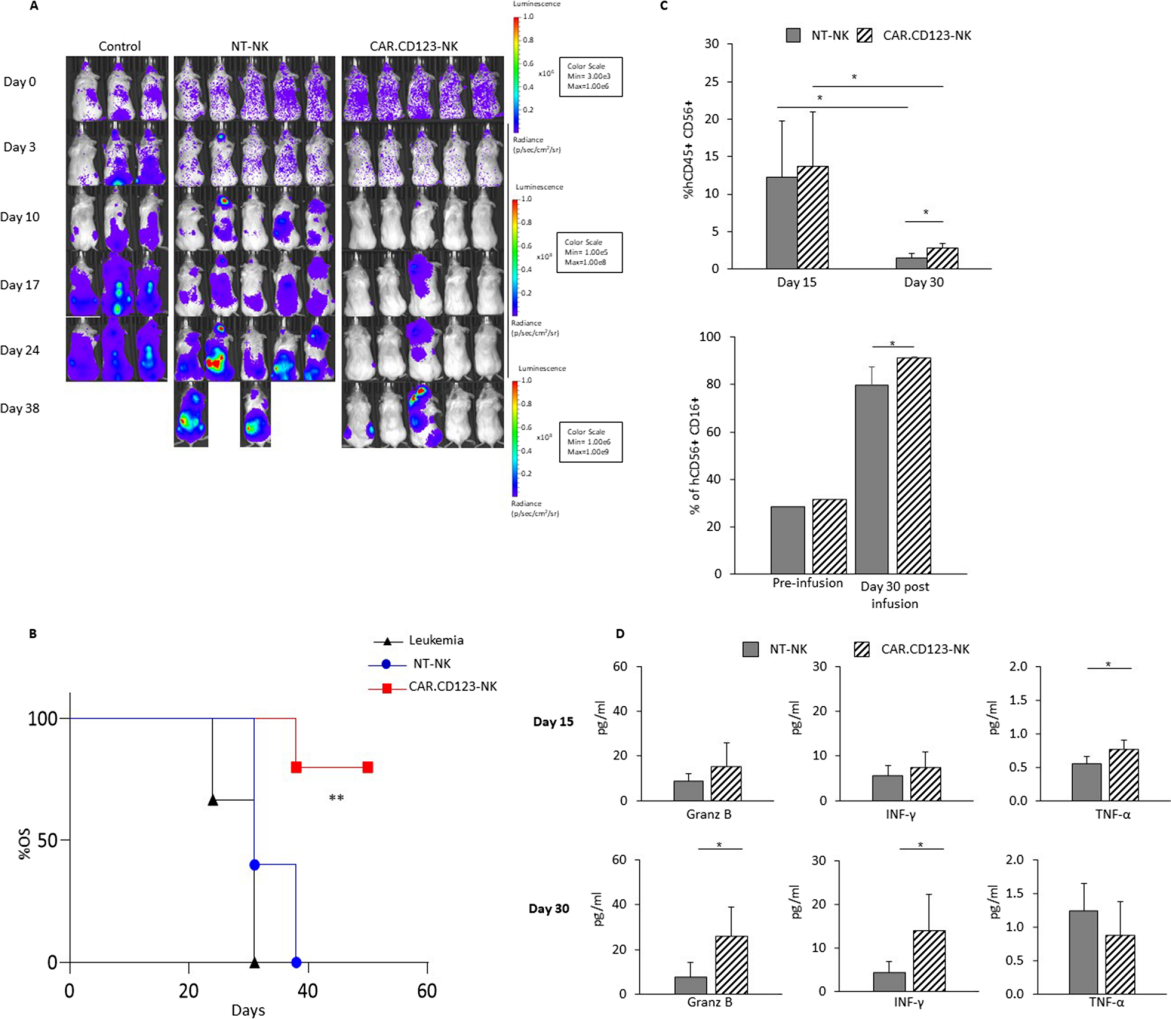
CAR.CD123-NK cells maintain efficacy and safety profile in an AML xenograft animal model based on hIL15-NOG mice. **A** hIL15-NOG mice were systemically engrafted with THP-1-FF-Luc.GFP cells and, at the time of leukaemia engraftment, treated or not with NT-NK and CAR.CD123-NK cells. Time course of in vivo bioluminescence imaging of the treated NOG mice. **B** Fifty-day probability of OS for NSG mice systemically infused with THP-1-FF-Luc.GFP cells after adoptive transfer of NT-NK (blue bars with circles) or CAR.123-NK cells (red bars with squares). The untreated control is represented by a black line with triangles. **C** Flow cytometry analysis of human NK cells in PB of mice (*n* = 5 mice receiving NK-NT; *n* = 5 mice receiving CAR.CD123-NK) performed at Day 15 and Day 30 after effector cell infusion. Plots show the percentage of hCD45^+^ hCD56^+^ hCD3^−^ cells gated on the total cells present in PB of the mice (left panel) and as the percentage of hCD45^+^ hCD16^+^ cells of the total hCD45^+^ hCD56^+^ CD3.^−^ cells present in PB of the mice (right panel). Data are shown as average ± SD. *p < 0.05. **D** Cytokine concentration was measured in the plasma of mice bearing THP-1-FF-Luc.GFP cells and treated with NT-NK (grey bars) and CAR.CD123-NK (black line bars) cells at Day 15 and at Day 30 after effector cell infusion. Granz B, IFN-γ and TNF-α were measured by ELISA assay, and data are shown as average ± SD. *p < 0.05, **p < 0.01

**Fig. 7:**
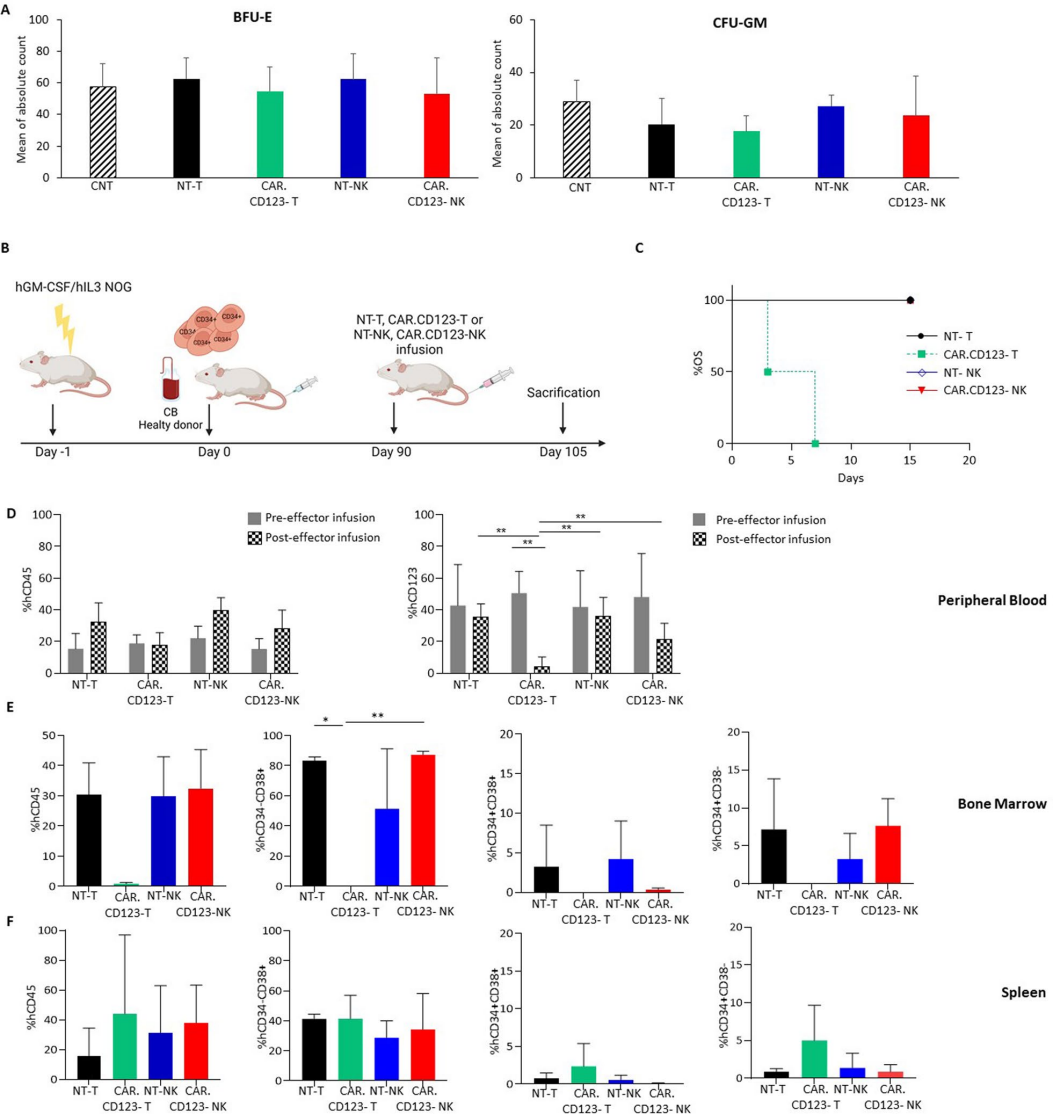
In vitro and in vivo on-target off-tumour effect of both CAR.CD123-T and CAR.CD123-NK cells against hematopoietic precursors. **A** Colony-forming unit (CFU) assay was performed with CD34^+^ cells derived from BM samples of 3 HDs after a short-term 4-h coculture with media alone (control condition, CNT; black line bars), un-transduced T cells (NT-T; black bars), CAR.CD123-T (green bars), un-transduced NK cells (NT-NK; blue bars) or CAR.CD123-NK cells (red bars). BFU-E and CFU-GM colonies were counted after 14 days. Results are shown as average of absolute count ± SD. **B** Schematic representation of humanized mouse model of hGM-CSF/hIL3 NOG (hu-NOG) mice engrafted with CD34^+^ cells derived from cord blood (CB) and infused with effector cells. **C** 15-Day probability of OS for humanized mouse model engrafted with adoptive transfer of NT-T (black line with circle), CAR.CD123-T (dotted green line with squares), NT-NK (blue line with rhombus) and CAR.CD123-NK (red line with triangles) cells. **D** Flow cytometry analysis of hCD45 cells in PB of hu-NOG mice before (grey bars) and (squared pattern bars) after 4 days from the effector cells infusion. Graphs show the percentage of hCD45^+^ cells on the total mononuclear cells present in PB (left panel), and the percentage of hCD123^+^ cells on the total hCD45^+^ cells present in the PB samples (right panel). Data are shown as average of ± SD. **E**–**F** Flow cytometry analysis of human hematopoietic cell quantification in tibia BM (E) and spleen (F) of hu-NOG mice receiving NT-T (black bars), CAR.CD123-T (green bars), NK-NT (blue bars) or CAR.CD123-NK (red bars). Graphs report the percentage of hCD45^+^ cells on the total mononuclear cells present in the investigated tissue (first panel), the percentage of hCD34^−^CD38^+^ cells on the total hCD45^+^ cells (second panel), the percentage of hCD34^+^CD38^+^ cells on the total hCD45^+^ cells (third panel), and the percentage of hCD34^+^CD38^−^ cells on the total hCD45.^+^ cells (fourth panel). Data are shown as average ± SD. *p < 0.05, **p < 0,001

The anti-leukaemia activity of CAR.CD123-NK cells were compared to the anti-leukaemic effect of standard T effector cells genetically modified with the same CAR.CD123 construct in an AML xenograft immune-deficient mouse model. NSG mice were intravenously engrafted at Day -3 with 5 × 10^5^ THP-1 cells/mouse; THP-1 cells had been genetically modified to express luciferase (THP-FF-Luc.GFP). As shown in Fig. 5A, mice showing leukaemia engraftment were treated on Day 0 and Day 7 with 5 × 10^6^/mouse effector cells (NT-T, CAR.CD123-T, NT-NK or CAR.CD123-NK cells). Bioluminescent imaging (BLI) was used to measure the AML burden over time (Fig. 5B–C). As expected, bioluminescence increased rapidly in mice treated with both NT-T and NT-NK cells (Fig. 5B–C). Notably, CAR.CD123-NK cells induced kinetics of leukaemia control similar to those of CAR.CD123T cells (Fig. 5B–C). These data were also corroborated by the overall survival (OS) analysis of the mice in each of the considered cohorts. In particular, leukaemia-engrafted mice treated with either CAR.CD123-NK cells or CAR.CD123-T cells showed a significantly improved OS compared to mice treated with control conditions (NT-T or NT-NK cells; Fig. 5D). Remarkably, no difference was observed in the OS of mice treated with either CAR.CD123-NK cells or CAR.CD123-T cells. However, despite CAR.CD123-NK cells exerting significant anti-leukaemia activity, we did not observe effector cells expanding in the PB of treated mice. Therefore, we decided to use a NOD.Cg-Prkdcscid Il2rgtm1Sug Tg (CMV-IL2/IL15) (hNOG-IL15) xenograft mouse model in which the human IL-15 cytokine is constitutively produced, mimicking the patient’s environment after lymphodepletion [36]. Thus, hNOG-IL15 mice were engrafted with THP-FF-Luc.GFP cells and then treated with a single dose of 20 × 10^6^ CAR.CD123-NK cells and control NT-NK cells (Additional file 1: Fig. S7A). Additionally, in this animal model, CAR.CD123-NK cells were able to significantly control leukaemia progression compared to NT-NK cells in the control mouse cohort (Fig. 7A and Additional file 1: Fig. S7B), translating into a significantly prolonged OS of the mice (median OS of 31 days for mice treated with NT-NK cells, range 31–38; OS timing undefined for mice receiving CAR.CD123-NK cells; *p* = 0.009; Fig. 6B). Moreover, at Day 15 after the infusion of the effector cells, we observed a great expansion of both NT-NK and CAR.CD123-NK cells in the PB of mice (Fig. 6C), with CAR.CD123-NK cells persisting at significantly higher levels than NT-NK cells at Day 30 (Fig. 6C). Concurrently, in this latter animal model, we were also able to detect inflammatory cytokines in the plasma of treated mice (Fig. 6D) with respect to the null level observed in the NSG model (Additional file 1: Fig. S6).

In particular, the plasma concentrations of Granz B, IFN-γ and TNF-α were detected at Day + 14 and Day + 30, with significantly higher levels of Granz B and IFN-γ at Day + 30 in mice treated with CAR-NK cells than in mice receiving NT-NK cells (Fig. 6D, for both Granz B and IFN-γ *p* < 0.05). These data corroborate the higher anti-leukaemia activity of CAR.CD123-NK cells than NT-NK cells. Notably, in this model, it was also possible to study the phenotypic features of the expanded NK cells in terms of the regulation of CD16, CD57 and PD1 markers. Indeed, we observed that NK cells expanded in vivo in the animal model with constitutive production of human IL15 and significantly upregulated the antibody-dependent cellular cytotoxicity (ADCC) Fcγ receptor CD16 (Fig. 6C), the differentiation marker CD57 and the checkpoint inhibitor receptor PD-1 (Additional file 1: Fig. S7C) [37].

### CAR.CD123-NK cells did not lead to the ablation of normal human haematopoiesis in a humanized animal model

The expression of CD123 on normal HCs, including circulating B cells, myeloid progenitors, dendritic cells, and megakaryocytes, raises concerns regarding potential on-target off-tumour effects. In addition to the demonstration that CD123 is expressed at low levels on normal BM haematopoietic precursors of HDs, we sought to investigate the effect of CAR.CD123-NK cells on normal haematopoiesis, in comparison with that of CAR.CD123-T cells, which have already been reported to have significant cytotoxicity against normal HPSCs in vivo [38]*.*

To evaluate the potential haematopoietic toxicity of the anti-CAR.CD123-NK cells, we exposed CD34^+^ cells derived from the BM of HDs to CAR.CD123-NK and CAR.CD123-T cells for 4 h. In vitro, we did not observe any effect on the formation of CFU-GM and BFU-E colonies under any of the tested conditions (Fig. 7A). We then investigated the potential in vivo toxicity of CAR.CD123-NK cells on normal CD34^+^ HSPCs in a more comprehensive humanized mouse model (Fig. 7B). For this purpose, hGM-CSF/hIL3 NOG (hNOG-3GM) mice were engrafted with human umbilical cord blood-derived CD34^+^ HSCs after sublethal irradiation, and at 10 weeks post-transplantation, mice were infused with CAR.CD123-T, CAR.CD123-NK or unmodified T or NK cells (Fig. 7B). Five days after the injection of effector cells, all the mice infused with CAR.CD123-T cells were dead, developing acute toxicity; in contrast, mice treated with CAR.CD123-NK cells did not show associated toxicity, as indicated by OS data, with 100% of the mice surviving at Day 15 (end of the experiment) (Fig. 7C).

To better deconvolute the mechanism underlying the observed acute toxicity, we performed an experiment killing the above-described humanized mice early (4 days after the effector cell infusion) and harvesting and analysing their PB and the most relevant haematopoietic organs, including the tibia, BM and spleen.

We observed that CAR.CD123-T cells significantly reduced the overall percentage of circulating CD123^+^ cells in PB, whereas this effect was only marginally found in mice receiving CAR.CD123-NK cells (Fig. 7D). Nevertheless, total myeloid CD33^+^ cells, but not total CD3^+^ or CD19^+^ cells, were significantly reduced in the cohort of mice receiving CAR.CD123-T cells compared with mice treated with CAR.CD123-NK cells (Additional file 1: Fig. S8). Moreover, mice treated with CAR.CD123-T cells showed severe BM aplasia, with almost null CD45^+^ cells as well as Lin^−^hCD34^+^CD38^−^, Lin^−^hCD34^+^CD38^+^ and Lin^−^hCD34^−^CD38^+^ HSCs detected in the BM (Fig. 7E). Ablation was not observed in the spleen (Fig. 7F), in which the staminal compartment of Lin^−^hCD34^+^CD38^−^ cells was less represented. Importantly, aplasia was not observed at all in the mice infused with control NK cells, control T cells or CAR.CD123-NK cells (Fig. 7E–F).

### Evaluation of the toxicity profile of CAR.CD123-NK cells versus human endothelial tissue: in vivo model

It has been previously reported that the CD123 antigen is expressed on endothelial cells and that these cells are specifically recognized and lysed by CAR.CD123-T cells [39]. Moreover, the expression of CD123 is upregulated on endothelial cells by both INF-γ and TNF-α, cytokines known to be significantly associated with CRS in patients treated with CAR T cells [9]. In light of these findings, we sought to define the toxicity profile of CAR.CD123 cells in a comprehensive animal model in which we developed a human endothelial tissue.

**Fig. 8:**
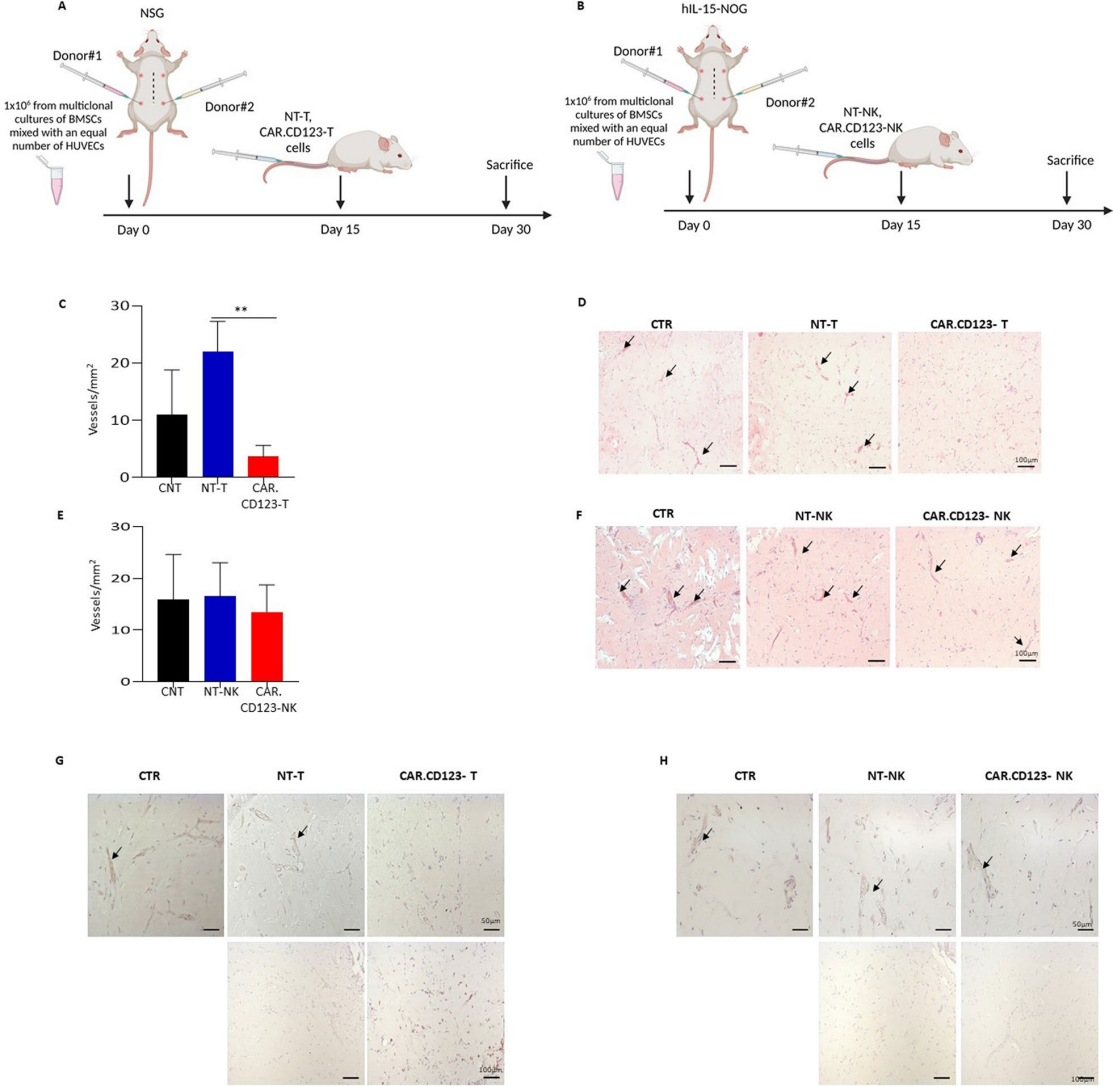
CAR.CD123-NK cells do not target the human endothelial, preserving the vessels integrity in the murine model. **A**–**B** Schematic representation of angiogenesis assay in NSG mice engrafted at Day 15 with NT-T or CAR.CD123-T and hIL-15NOG mice engrafted at Day 15 with NT-NK or CAR.CD123-NK cells. Mice were killed at 30 days post-Matrigel injection. **C** The number of vessels per mm^2^ was reduced in CAR.CD123-T cells-bearing NSG mice (red column) as compared to control (mice that were not infused with effector cells, black column) or to mice receiving NT-T cells (blue column). Data are shown as average ± SD; **p < 0,001. **D** Haematoxylin and eosin-stained sections of Matrigel plugs showing the formation of an extensive network of capillary-like blood vessels in control and in NT-T infused mice, in contrast to CAR.CD123-T infused mice. **E** hIL-15NOG mice carrying Matrigel plugs were injected with NK-NT (blue column) or CAR.CD123-NK cells (red column), and no differences were observed in the vessels number compared to the control condition (CNT, black column). Data are shown as average of ± SD. **F** Haematoxylin and eosin-stained sections of Matrigel plugs derived from hIL15NOG mice infused with CAR.CD123-NK show no differences in the newly formed capillary network. **G** Representative images of immunohistochemical staining for CD123 (top panels) and CD45 (bottom panels) in samples derived from mice infused with NT-T or CAR.CD123-T cells. **H** Representative images of immunohistochemical staining for CD123 (top panels) and CD45 (bottom panels) in samples derived from mice infused with NT-NK or CAR.CD123-NK cells

In particular, BM stromal cells from HDs were implanted subcutaneously in Matrigel, along with human umbilical vein endothelial cells (HUVECs), on the right and left flanks of mice, resulting in the formation of an extensive network of capillary-like nascent blood vessels (BVs) 2 weeks after implantation [33]. We first engrafted the endothelial model in standard NSG mice to test the safety profile of effector T cells (10 × 10^6^ CAR.CD123-T cells or NT-T cells) that were infused 2 weeks after implantation. Animals were killed to collect the transplanted endothelium (Fig. 8A). Whereas the controls developed a dense network of capillary-like nascent BVs, as assessed by haematoxylin/eosin staining and the number of vessels/mm2 (Fig. 8C–D), we observed a significant reduction in the number of vessels per mm2 in mice receiving CAR.CD123-T cells (*p* = 0,001). To test the effect of CAR.CD123-NK cells on endothelial cells, we implanted the endothelial cell construct in a more challenging hNOG-IL15 mouse model that allows for NK-cell expansion and persistence in vivo. In this model, 20 × 10^6^ CAR.CD123-NK cells and NT-NK cells were infused (Fig. 8B), and mice were killed after 2 weeks to collect endothelial tissues. As reported in Fig. 8E–F, we did not observe any differences in terms of the number of vessels/mm^2^ in mice treated with CAR.CD123-NK cells or NT-NK cells. Last, we observed a high density and frequency of human CD45^+^ cells in Matrigel sections of endothelial grafts, proving the better ability of CAR T cells than NT-T cells to infiltrate and destroy vessels. In contrast, we did not observe CD45^+^ cell infiltration of the endothelial tissues of the mice treated with either NK-NT or CAR-NK cells (Fig. 8G–H), although we were able to demonstrate the persistence of NK cells in the PB of hIL15-NOG mice (Additional file 1: Fig. S9).

## Discussion

In the field of CAR-cell-based therapies, recent preclinical and clinical studies have shown that primary NK cells can be genetically modified to express a CAR in a feeder-free condition [22] and that CAR-NK cells are safe and potentially very effective against r/r CD19-positive cancers (including non-Hodgkin’s lymphoma and chronic lymphocytic leukaemia) [25]. Many clinical data concur in demonstrating a clinical benefit for patients affected by B cell lymphoid malignancies treated with CAR.CD19 T cells [40, 41], but while CD19 is well known to be a good target for CAR T cell-based therapy, in AML, the application of single-targeting approaches remains difficult, as none of the known antigens are exclusively expressed on AML cells, raising the concern of potential severe on-target off-tumour toxicity [12, 42]. Moreover, although CAR T cells have been extensively investigated in preclinical and clinical models of AML, thus far, across all AML-reported clinical trials, only one-quarter of AML-treated patients achieved some objective responses [4]. This observation prompted the need to further optimize adoptive cell therapies in these patients.

To the best of our knowledge, there are limited preclinical studies [43, 44], and currently, only 4 active clinical trials (NCT05215015, NCT04623944, NCT05247957, NCT05008575) are testing CAR-NK cells in AML patients; in addition, there is a single completed anti-AML CAR.NK-cell trial with evaluable data (NCT02944162) [45] showing that CAR.CD33-NK92 cell infusion was safe but, unfortunately, associated with minimal anti-leukaemia efficacy. Of note, the use of NK-92 cells has the great advantage of an allogeneic cell line that it is easy to expand and transduce in vitro, but, as also required by current regulation, to avoid in vivo overgrowth, irradiation is mandatory before infusion [46].

In this scenario, we have already proven that PB is a suitable and valid source of allogeneic cells for the generation of CAR-NK-cell products using a feeder-free clinical-grade protocol [22]. In this paper, we sought to evaluate, in a preclinical model, the safety and efficacy of a novel allogeneic off-the-shelf product based on CAR-NK cells for the treatment of AML.

In particular, the heterogeneity of the different leukaemia subtypes and the similarities between normal HSCs and LSCs render the identification of an appropriate target antigen difficult. Interleukin-3 receptor alpha (IL-3RA; CD123) is overexpressed on the AML blasts and LSCs of patients with poor prognoses [47]. Remarkably, all the most frequent phenotype (immature, granulocytic and monocytic) and genetic AML subgroups express CD123 [5, 48, 49].

We initially analysed the cell surface expression of CD123 on blasts from paediatric patients diagnosed at our institution, proving that CD123 is expressed in the majority of AML cases in the total blast population and in the fraction of CD34^+^ cells, which is presumed to contain LSCs. Moreover, we reported a differential expression of CD123 between HSCs and LSCs, and this finding provided support for the rationale to develop an adoptive cell therapy based on CAR-NK cells targeting CD123. Remarkably, the LSC compartment is characterized not only by a higher percentage of CD123^+^ cells but also by a significantly higher MFI of the target antigen than HSCs. These data are of particular relevance, since the negative impact of the CD34^+^ CD38^−^ CD123^+^ cell population on the disease-free survival and OS of AML patients has already been documented [50], with this cell population being resistant to chemotherapy [35]. Thus, targeting CD34^+^CD123^+^ cells could represent an optimal strategy to eradicate AML compared to approaches based on CAR-NK cells targeting CD38 [43].

In the context of normal haematopoietic cells, a low level of CD123 expression was observed across all myeloid maturation stages, with a higher MFI of CD123 in the most differentiated CD38^+^CD34^−^ cells. This observation, together with the data already present in the literature on this topic [51], led us to pay particular attention to exploring the on-target off-tumour effect and safety profile of a CAR product targeting CD123.

We first obtained proof-of-concept of the anti-leukaemia activity of NK cells genetically modified to express the second-generation CAR.CD123.41bb.CD3z construct. In particular, allogeneic CAR.CD123-NK cells mediate significant cytotoxicity against the CD123^+^ cell line and primary AML blasts, providing encouraging data that the innate activity of NK cells could be optimized by the addition of CAR to overcome the leukaemia resistance already largely described in patients receiving NK cells through adoptive transfer [52]. With the aim of developing a therapy that could target LSCs in AML, we deeply investigated the anti-leukaemia activity of CAR.CD123-NK cells on all CD123^+^ subsets of AML primary samples, including the most mature CD38^+^ CD34^−^, the intermediate CD38^+^CD34^+^ and the undifferentiated CD38^−^CD34^+^ leukaemic cells. We proved that CAR.CD123-NK cells were not only highly activated against AML blasts, as shown by the production of pleiotropic cytokines, but also, and most importantly, significantly eliminated CD123^+^ blasts after 6 days of in vitro culture.

The anti-leukaemia activity of CAR.CD123-NK cells was also confirmed in a xenograft mouse model in which NSG mice were engrafted with a human AML CD123^+^ cell line. In particular, we observed higher anti-leukaemia activity of CAR.CD123-NK cells than NT-NK cells, whereas no significant differences in leukaemia control were found in comparison with T cells genetically modified with the same CAR.CD123. Moreover, although the OS of mice treated with CAR.CD123-T and CAR.CD123-NK cells was not significantly different, we observed the development of a xenograft reaction in the CAR.CD123-T cell cohort, leading to the death of all the treated mice, whereas CAR.CD123-NK cells did not induce a xenograft reaction at all. However, in line with the CAR-NK model that we have previously developed in BCP-ALL [22], we did not observe CAR-NK cells in the PB of the treated mice at Day 14, nor did we observe detectable levels of human cytokines, including IFN-γ, IL-2 and TNF-α. Thus, we investigated the behaviour of CAR.CD123-NK cell activity in a NSG mouse model constitutively expressing hIL-15 (hIL15-NOG) [36], which could better mimic the cytokine *milieu* of patients treated with standard lymphodepletion before the infusion of CAR T cells [53]. In particular, IL-15 has been reported to support the persistence and in vivo expansion of NK cells, although CAR.CD123-NK cells engineered to secrete IL-15 were associated with systemic toxicity in an animal model [54].

The AML-engrafted hIL15-NOG murine model was particularly relevant not only to confirm the significant anti-leukaemia activity of CAR.CD123-NK cells but also to detect NK cells in the PB of mice at least up to 30 days after infusion in the absence of any toxicity. This model allowed us to characterize the circulating CAR-NK cells for their expression of CD16, CD57 and PD-1; we observed a shift from the CD56^high^CD16^low^ to CD56^low^CD16^high^ phenotype, with increasing expression of the maturation marker CD57. In particular, both NT- and CAR-NK cells overexpressed the ADCC receptor CD16 after in vivo expansion, this finding providing support to the possibility of developing a combinational strategy between adoptive NK cells and ADCC-based monoclonal antibodies [23]. With regard to PD1 expression, CAR.CD123-NK cells significantly upregulated this antigen in vivo compared to NT-NK cells. The role of PD1 in NK cells is still largely debated and controversial [55]. In T cells, PD1 is a well-known checkpoint inhibitor that, when expressed in combination with other antigens, including TIM-3 and LAG-3, characterizes exhausted T cells, although it has also been associated with activated effector cells. In the field of adoptive cell therapy, PD1 has been described to be overexpressed in active and cytotoxic CAR T cells [56]. Further functional studies need to be performed to better define the significance and impact of PD1 overexpression in in vivo expanded CAR-NK cells.

Once the efficacy was proven, we next sought to define the safety profile of the CAR.CD123-NK cell product. In particular, CD123 targeting is strongly debated for the risk of inducing prolonged and profound myelosuppression due to the killing of healthy haematopoietic cells; therefore, we analysed the effect of CAR.CD123-NK cells on HD-derived BM CD34^+^ cells, observing no substantial reduction in CFU-GM or BFU-E colony formation when haematopoietic progenitors were cocultured in vitro for 4 h with either CAR.CD123-NK or T cells. Nevertheless, a significantly different safety profile between CAR.CD123-NK and CAR.CD123-T cells was observed in the animal model in which NSG-3SG mice were sublethally irradiated and humanized through the infusion of CD34^+^ cells from cord blood. CAR.CD123-T cell-treated mice experienced acute, severe and fatal haematologic toxicity leading to death in the first week after CAR.CD123-T cell infusion. A deeper evaluation of this toxicity revealed that mice infused with CAR T cells developed severe myeloablation in the BM, with a significant decrease in the number of total hCD45^+^ cells and, more specifically, in the Lin^−^hCD34^+^CD38^+^ and Lin^−^hCD34^−^CD38^+^ subsets and the Lin^−^hCD34^+^CD38^−^ haematopoietic stem cell compartment. In contrast, mice infused with CAR.CD123-NK cells did not develop any sign of toxicity or any ablation of cell subtype in the most relevant murine haematopoietic tissues. The differential behaviour of humanized mice receiving CAR T or CAR-NK cells could not be explained by the different allograft profile of the two effectors, since no toxicity at all or reduction in the haematopoietic compartment was observed in mice infused with control un-transduced T cells, underlying a relevant impact on the safety of CAR.CD123 when expressed on T cells. All these findings are consistent with those reported in several preclinical studies about severe cytopenia caused by CAR.CD123-T cells [12] and support the evidence of a differential safety profile of CAR.CD123-NK cells.

Endothelial cells express the CD123 antigen at low levels [39], and previous studies have already reported that its upregulation, after INF-γ and TNF-α in vivo exposure, drives specific recognition and cytotoxicity by CAR.CD123-T cells [9]. Thanks to an in vivo model, in which we recapitulated human endothelial tissue in mice, we were able to prove for the first time that CAR.CD123-NK cells and CAR.CD123-T cells also exert differential toxicity in this context. In particular, human endothelial cells grown on a scaffold were implanted in standard NSG mice, and after human endothelial cells were engrafted for 4 weeks, mice were infused with CAR.CD123-T cells. The histological analysis revealed that the vessel count was significantly reduced in these mice compared to control mice receiving unmodified T cells. Moreover, significant T cell infiltration has been documented in the injured tissue. To evaluate the toxicity of CAR.CD123-NK cells on the endothelium, we challenged the model in hIL15-NOG mice, since we have already proven that CAR-NK cells could persist for at least 30 days. Histological analysis of Matrigel plugs of human endothelial cells engrafted in hIL15-NOG mice infused with CAR.CD123-NK cells did not reveal a significant reduction in vessel growth or number with respect to the control condition of mice infused with unmodified NK cells. In this case, the human endothelial tissue did not show a significant infiltration of NK cells (assessed as human CD45^+^ cells), whereas at the time of mouse killing, CAR-NK cells and NK cells were still detectable in the PB.

## Conclusions

In conclusion, our studies provide novel insights into the field of adoptive cell therapy for AML, underlying the significant anti-leukaemia activity exerted by an allogeneic, off-the-shelf approach based on CAR.CD123-NK cells that represents a product that is equally as effective as CAR T cells but is characterized by a more favourable safety profile with regard to both haematopoietic and endothelial on-target off-tumour toxicity. To better assess the potency and safety of the developed CAR-NK cell product, we developed comprehensive animal models that can also be largely employed in the field of other innovative adoptive immunotherapies before the products enter early clinical trials.

## Supplementary Information

**Additional file 1.** Supplementary Materials.

## Data Availability

All data are available in the main text or in the supplementary materials. All reagents reported in the paper may be available for the scientific community, upon an agreement signed between the parties for their specific use.

## Abbreviations

AML: Acute myeloid leukaemia
FDA: Food and Drug Administration
ADCC: Antibody-dependent cellular cytotoxicity
BCP-ALL: B cell precursor acute lymphoblastic leukaemia
BMSCs: BM stem cells
BM: Bone marrow
BMMNCs: Bone marrow mononuclear cells
BC: Buffy coat
CAR.CD123: CAR targeting CD123
CAR: Chimeric antigen receptor
CR: Complete remission
CRS: Cytokine release syndrome
E:T: Effector/target cell ratio
eGFP-FFLuc: EGFP-firefly luciferase
EMA: European medicines agency
GvHD: Graft-versus-host disease
HSC: Haematopoietic stem cells
HD: Healthy donor
HSCT: Hematopoietic stem cell transplantation
HSC-MPP: Hematopoietic stem cells and multipotent progenitors
hHCs: Human haematopoietic cells
ICANS: Immune effector cell-associated neurotoxicity syndrome
IFN-γ: Interferon gamma
IL-3Rα: Interleukin-3 receptor alpha chain
LSC: Leukaemia stem cells
LMPP: Lymphomyeloid-committed progenitor
MSC: Mesenchymal stromal cells
NK: Natural killer
hNOG-IL15: NOD.Cg-Prkdcscid Il2rgtm1Sug Tg (CMV-IL2/IL15)
PBMCs: Peripheral blood mononuclear cells
rh-IL: Recombinant human interleukin
r/r AML: Relapsed or refractory AML
scFv: Single-chain variable fragment
St Dv: Standard deviation
TNF-α: Tumour necrosis factor alfa
VPs: Viral particles
