# Supplementary material for: Safe and effective off-the-shelf immunotherapy based on CAR.CD123-NK cells for the treatment of acute myeloid leukaemia
Source: J Hematol Oncol. 2022 Nov 5;15:163. doi: 10.1186/s13045-022-01376-3 (PMC9636687; doi:10.1186/s13045-022-01376-3)
Supplement: Supplementary file 1 — Additional file 1. Supplementary Materials. [file 13045_2022_1376_MOESM1_ESM.pdf]

Supplementary Materials

Flow-cytometry analysis of CD123 expression on primary BM cells from samples of HDs and AML patients.

Cell surface expression of CD123 was analysed on primary cells from AML or HDs using conjugated mouse anti-human CD123-BV421 mAb (BD Biosciences, USA). Cells were also stained with Live dead, CD34-BB700 (BD Biosciences, USA), CD10-BUV737 (BD Biosciences, USA), CD90-BV786 (BD Biosciences, USA), CD38-PeCy7 (BD Biosciences, USA), CD34RA-BUV395 (BD Biosciences, USA), CD133-PE (Miltenyi Biotec, Inc., San Diego, CA, USA), Anti-Human Lineage Cocktail- FITC (BD Biosciences, USA), humanCD45-V500 (BD Biosciences, USA) and murineCD45-BV605 (BD Biosciences, USA). Unstained and fluorescence minus one (FMO) controls were used to identify gating boundaries.
Cells were incubated with mAbs (30 min at 4°C in the darkness), then washed in 1x phosphate-buffered saline (PBS) and analysed in a FACS-Fortessa flow cytometer and data were analysed by FACSDiva software (Becton Dickinson).

Angiogenesis assay.
$1x10^6$ cells from multiclonal cultures of bone marrow stromal cells were suspended in 300ml ml of Matrigel Growth Factor-Reduced (BD Biosciences Labware) with an equal number of HUVECs (ATCC). The resulting cell suspension was injected in the subcutaneous tissue of the back of NSG or hIL-15NOG mice, previously anesthetized. Up to 4 transplants per mice were performed. After 15 Days post Matrigel injection, NSG mice were infused with $10x10^6$ NT-T or CAR.CD123-T cells, while hIL-15NOG and were infused with $20x10^6$ NT-NK or CAR.CD123-NK cells. Matrigel plugs were harvested after 30 Days and processed for histological analysis.

Immunohistochemistry studies.
Matrigel plugs were immediately fixed in 4% formaldehyde and processed for paraffin embedding. Four-μm thick paraffin sections were stained with haematoxylin and eosin for standard histological analysis. For immunolocalization studies a rabbit polyclonal anti-CD123 (*Invitrogen*, PA5-85146; 1:250 on) and a mouse monoclonal anti-CD45 (Dako, M070101-2, 1:100, on) for were used.
Before primary antibodies incubation, antigen retrieval was achieved with heat-mediated methods in sodium citrate buffer pH6. After primary antibodies incubation, slides were repeatedly washed in PBS and then they were incubated with biotin-conjugated swine anti-rabbit IgG (#P0217, Agilent, Santa Clara, CA, USA) or biotin-conjugated rabbit anti-mouse IgG (#P0260 Agilent, Santa Clara, CA, USA) for 30 minutes at room temperature in a humidified chamber. After washing in PBS, slides were incubated with streptavidin conjugated horseradish peroxidase (#P0397, Agilent, Santa Clara, CA, USA) for 30 minutes at room temperature. The peroxidase reaction was developed using DAB substrate kit (SK-4105, Vector Laboratories, Burlingame, CA, USA), for one to three minutes, depending on the primary antibody. Nuclei were counterstained with haematoxylin and sections were dehydrated and mounted using a xylene-based mounting medium. Brightfield light microscopy images were obtained using Zeiss Axiophot microscope (Carl Zeiss).

Microvessel density analysis.

Microvessels were quantified by evaluation of 4-10 randomly selected 10X fields of H&E stained sections taken from the transplants. Microvessels were identified as lumenal structures containing red blood cells and counted. Micro-vessels density was reported as the average number of red blood cell filled microvessels from

the fields analyzed and expressed as vessels/mm2. Values reported for each experimental condition correspond to the average values ± S.D obtained from at least 2 individual mice with 4 transplants each.

Supplemental Figures

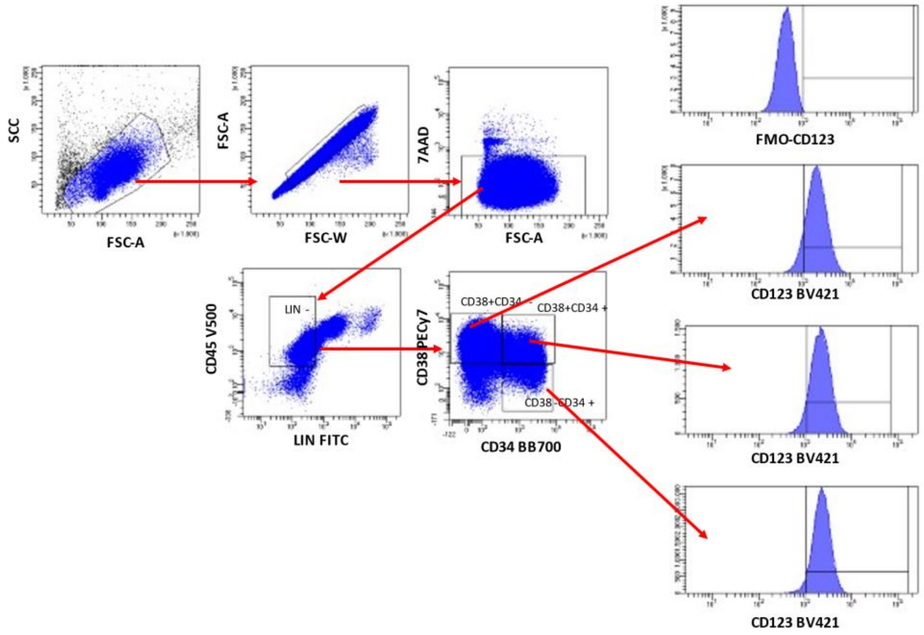

Supplemental Fig. 1. Gating strategy for the evaluation of CD123 positive cells among the differential maturation stages of human haematopoietic cells from AML patients. Gating strategy of a representative BM from AML patient to analyse CD123 expression on 3 different cell subsets: $CD38^{+}CD34^{-}$, $CD38^{+}CD34^{+}$ and $CD38^{-}CD34^{+}$.

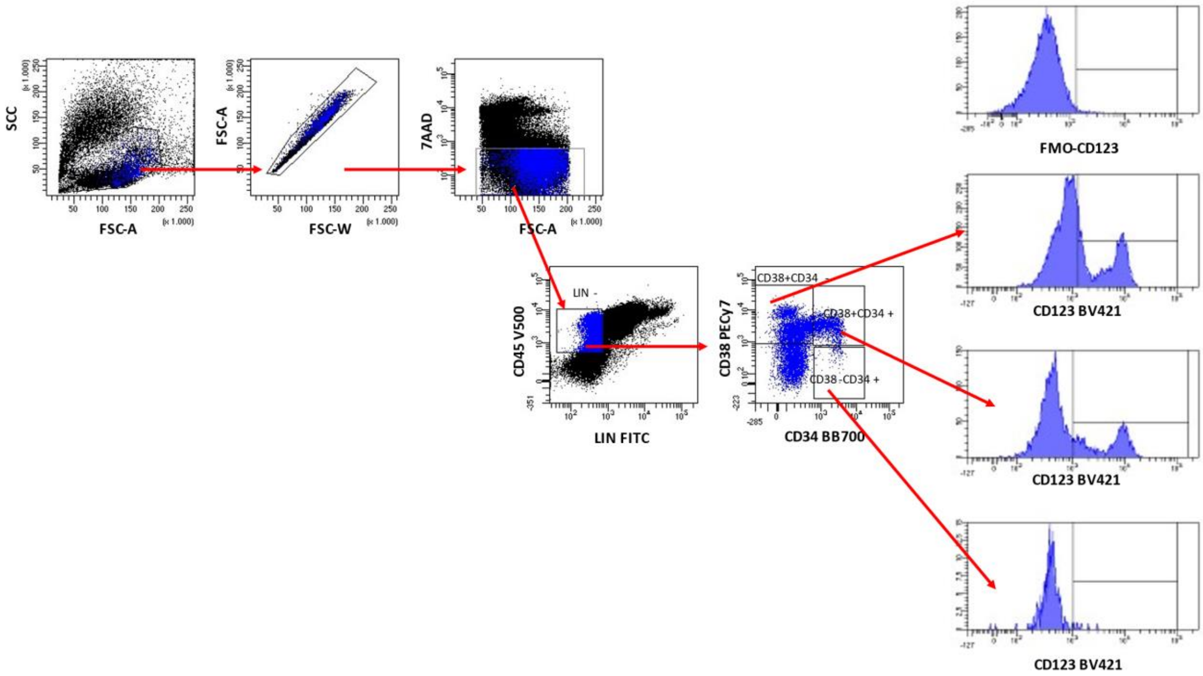

Supplemental Fig. 2. Gating strategy for the evaluation of CD123 positive cells among the differential maturation stages of human haemopoietic cells from HDs. Gating strategy of a representative BM from HD to analyse CD123 expression on 3 different cell subsets: $CD38^{+}CD34^{-}$, $CD38^{+}CD34^{+}$ and $CD38^{-}CD34^{+}$.

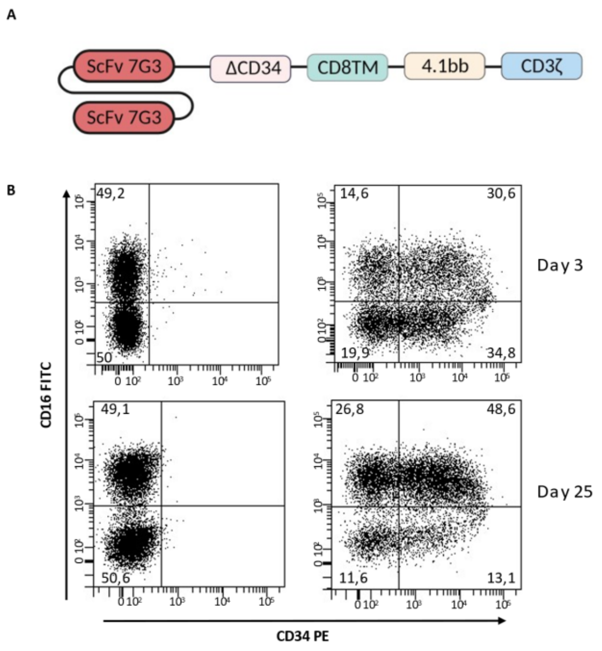

Supplemental Fig. 3. Retroviral vector carrying second generation CAR.CD123 for the gene modification of primary NK cells. (A) The expression cassette's illustration of a retroviral vector carrying second generation CAR.CD123 that includes the costimulatory domain of 4.1bb. The single-chain variable fragment (scFv) of CD123 derived from 7G3 monoclonal antibody, was cloned in frame with CD8aTM and the co-stimulatory domain of 4-1bb, as well as the CD3-zeta chain domain (ζ). As a trackable marker, we added a peptide derived from human CD34 (ΔCD34). (B) CAR.CD123 is efficiently expressed after retroviral transduction in both $CD56^{+}CD16^{-}$ and $CD56^{+}CD16^{+}$ NK cells. Plots are showing an exemplificative NK cell donor at Day 3 and Day 25 after transduction. CAR expression was assessed by the use of anti-CD34 (PE) mAb in combination with anti-CD56 (Pecy7) and anti-CD16 (FITC) mAbs.

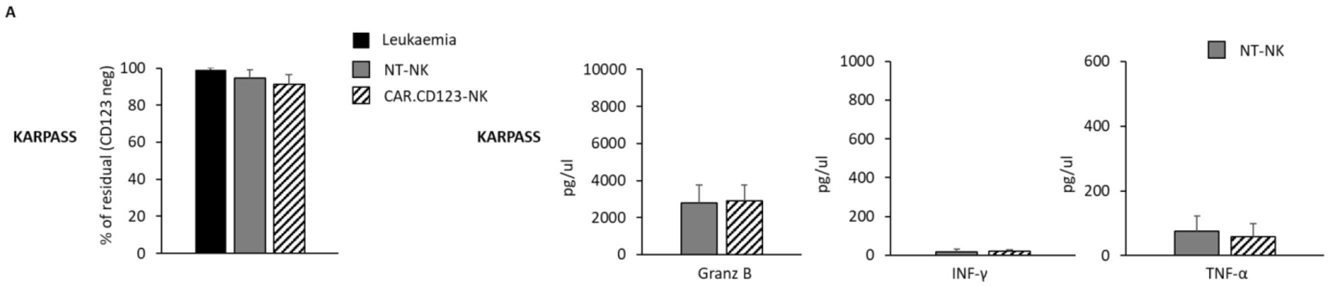

Supplemental Fig. 4. CAR.CD123-NK cells do not mediate killing in CD123 negative Karpas cell line. (A) Long-term cocultures were performed in 9 independent experiments, in which CD123negative lymphoma cell line (KARPASS) were cultured for 6 Days alone (black bar), with NT-NK (grey bars) or with CAR.CD123-NK cells (black line bars) at the E:T ratio of 1:1. Data are shown as average ± SD. (B) Granz B, IFN-γ and TNF-α were measured by ELISA assay in 24h culture supernatant of NT-NK (grey bars) or CAR.CD123-NK cells (black line bars) in response to CD123negative KARPASS cell line. Cytokine analysis was performed in 4 independent experiments and data are shown as average ± SD.

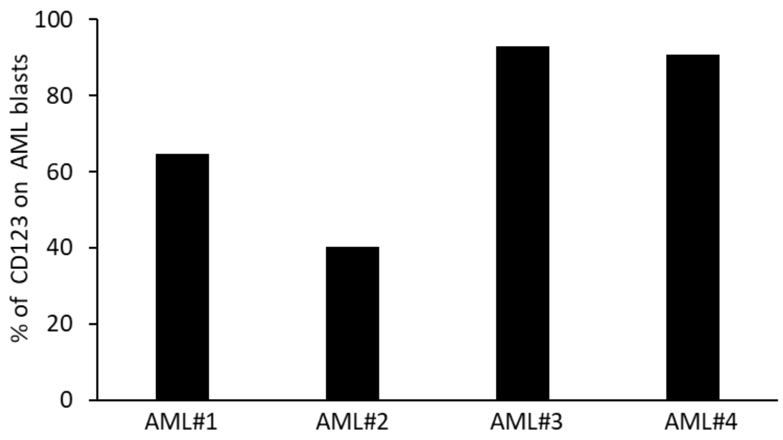

Supplemental Figure 5: CD123 epression on primary blasts from paediatric AML patients. Analysis of percentage of CD123 expression levels in primary fresh BM cells obtained from patients with AML used for *in vitro* studies

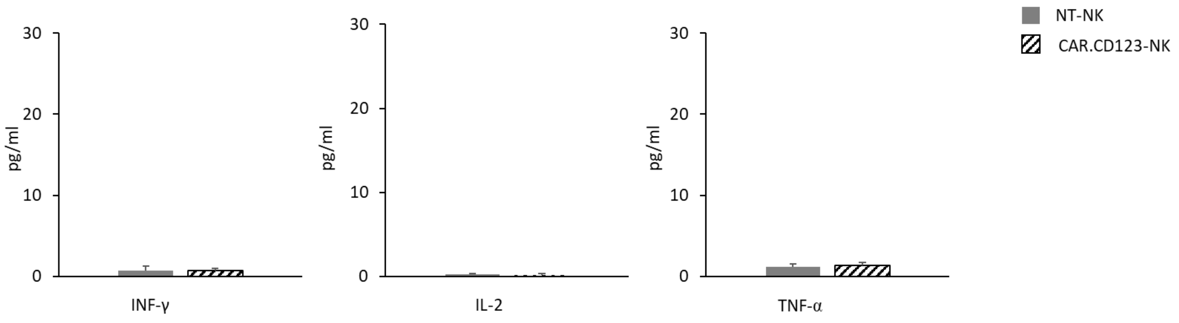

Supplemental Figure 6. Undetectable cytokine levels in the *in vivo* model of CAR.CD123-NK cells infused in AML xenograft NGS model. Cytokine concentration was measured in NSG mice serum bearing THP-1-FF-Luc.GFP cells and treated with NT-NK (grey bars) and CAR.CD123-NK (black line bars) cells at Day 14 after second effector cell infusion. IFN-γ, IL-2 and TNF-α were measured by ELISA assay and data are shown as average ± SD.

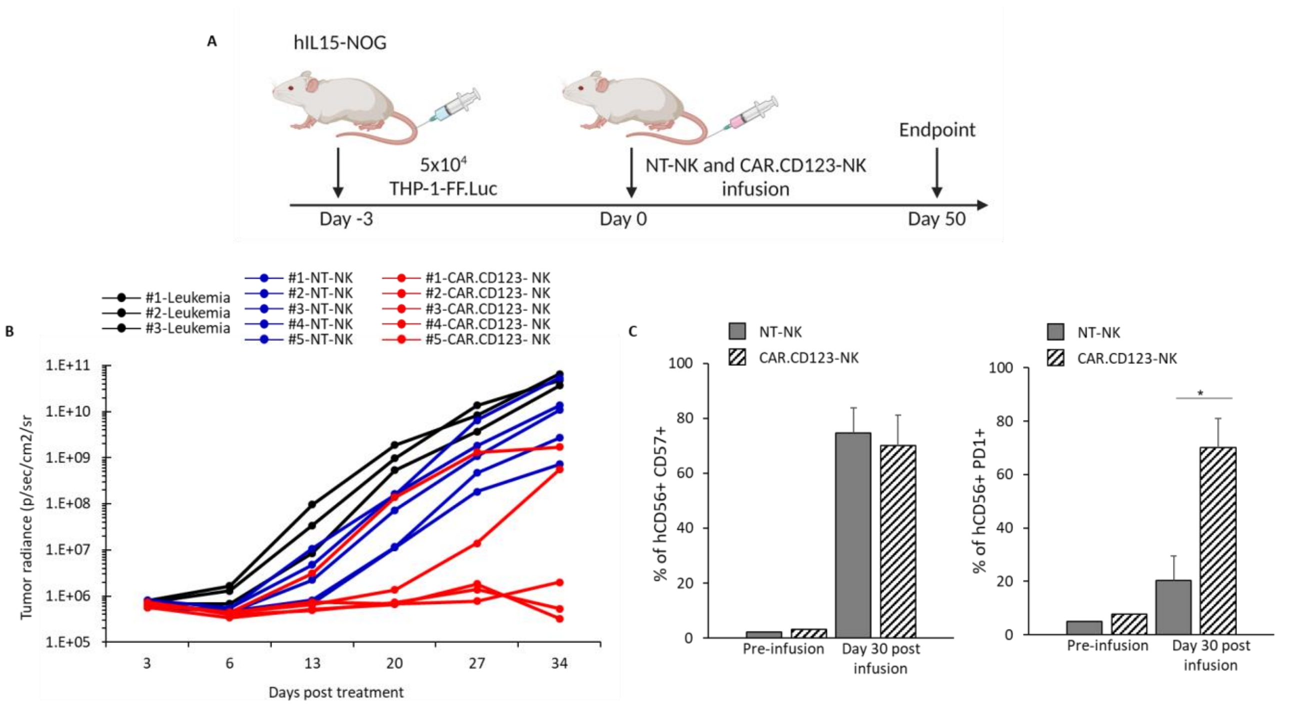

Supplemental Figure 7. AML xenograft animal model based on hIL15-NOG mice to evaluate efficacy and safety of CAR.CD123-NK cells. (A) Illustration of experimental setting in which hIL15-NOG mice were systemically infused with THP-1-FF-Luc.GFP cells and treated with a single dose of NT-NK or CAR.CD123-NK cells. (B) Graph shows bioluminescence analysis of each leukemia bearing mice treated with NT-NK (blue line) and CAR.CD123-NK (red line) cells or untreated control (black line). (C) Flow cytometric analysis of $hCD56^{+}CD57^{+}$ (on the left) and $hCD56^{+}PD1^{+}$ cells in PB of hIL15-NOG mice engrafted with THP-1 leukaemia cells and treated with NK-NT (black line), CAR.CD123-NK (blue line) at Day 30, in comparison to the effector cell characterization before the infusion in the mice. Data are shown as average ± SD. *$p < 0.05$.

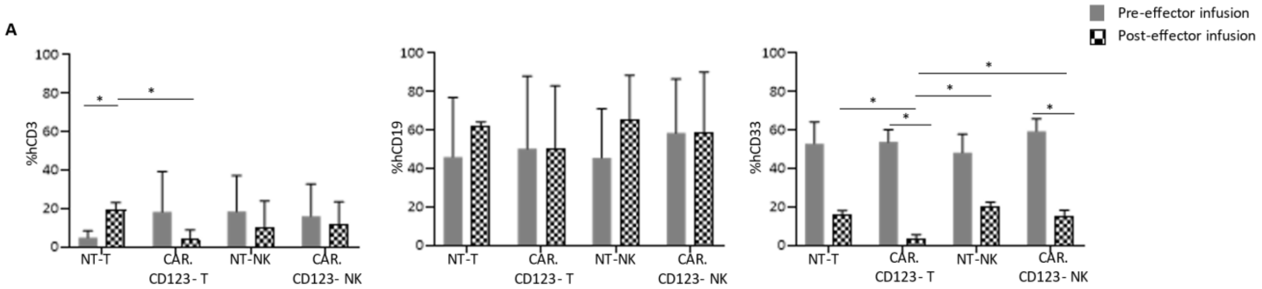

Supplemental Figure 8. ***In vitro*** **and** ***in vivo*** **on-target off-tumour evaluation of both CAR.CD123-T and CAR.CD123-NK cells against hematopoietic precursors.** (A) Evaluation of the percentage of CD3, CD19 and CD33 cells analysed in PB of hu-NSG mouse model, gating on hCD45 cells. Grey bars represent the percentage of human haematopoietic cells before the effector cell (NT-T and CAR.CD123-T or NK-NT and CAR.CD123-NK) infusion, while the squared pattern bars show the percentage of CD3, CD19 and CD33 after effector infusion. t-test was applied for statistical analysis and data are shown as average ± SD. *$p < 0.05$.

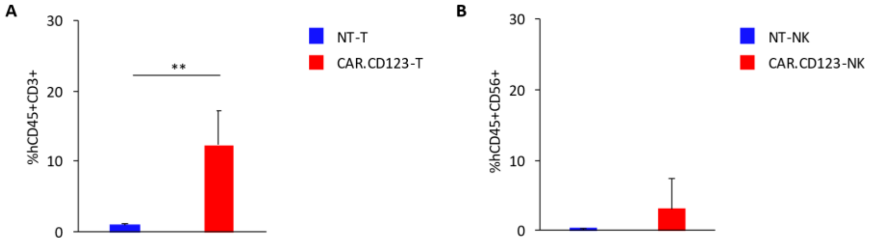

Supplemental Figure 9. Evaluation of CAR.CD123-T and CAR.CD123-NK cell persistence in the *in vivo* model of human endothelium. (A) Analysis of hCD45$^+$CD3$^+$ cells on Day 30 in PB of NSG leukaemia bearing mice treated with NT-T and CAR.CD123-T cells. (B) Analysis of hCD45$^+$CD56$^+$ cells on Day 30 in PB of hIL15-NOG leukaemia bearing mice treated with NT-NK and CAR.CD123-NK. t-test was applied for statistical analysis and data are shown as average ± SD $**p < 0.01$.
